# CoMPaseD: advanced planning of proteomic experiments aiming to identify small proteins

**DOI:** 10.1093/femsml/uqaf043

**Published:** 2026-01-06

**Authors:** Jürgen Bartel, Philipp T Kaulich, Borja Ferrero-Bordera, Rick Gelhausen, Rolf Backofen, Andreas Tholey, Sandra Maaß

**Affiliations:** Department of Microbial Proteomics, Institute of Microbiology, Center of Functional Genomics of Microbes, University of Greifswald, 17489 Greifswald, Germany; Systematic Proteome Research & Bioanalytics, Institute for Experimental Medicine, Christian-Albrechts-Universität zu Kiel, 24118 Kiel, Germany; Department of Microbial Proteomics, Institute of Microbiology, Center of Functional Genomics of Microbes, University of Greifswald, 17489 Greifswald, Germany; Department of Molecular Chronobiology, Institute of Medical Psychology, Medical Faculty, LMU Munich, 80336 Munich, Germany; Bioinformatics Group, Department of Computer Science, University of Freiburg, 79110 Freiburg, Germany; Bioinformatics Group, Department of Computer Science, University of Freiburg, 79110 Freiburg, Germany; Signalling Research Centre CIBSS, University of Freiburg, 79104 Freiburg, Germany; Systematic Proteome Research & Bioanalytics, Institute for Experimental Medicine, Christian-Albrechts-Universität zu Kiel, 24118 Kiel, Germany; Department of Microbial Proteomics, Institute of Microbiology, Center of Functional Genomics of Microbes, University of Greifswald, 17489 Greifswald, Germany

**Keywords:** proteomics, alternative protease, small proteins, experiment design, software, protein digestion

## Abstract

In proteome studies, the application of alternative proteases, exclusively or in addition to trypsin, often increases protein sequence or proteome coverage. It has recently been shown that, in particular, the analysis of small proteins benefits from such multi-protease approaches. However, selecting the most optimal combination of proteases either requires laboursome experiments or the decision of an experienced user, which might be biased. In this manuscript, we present a protease score that enables the objective comparison of multiple-protease digestions and a Python-based tool named CoMPaseD (Comparison of Multiple Protease  Digestions), which utilizes Monte-Carlo simulations to predict this score for a user-defined set of proteases and any combination of these. By analysis of the small proteomes of the two model organisms *Bacillus subtilis* and *Methanosarcina mazei* with five proteases and different experimental setups, we demonstrate a good correlation between experimentally derived and predicted scores. This highlights the broad applicability of CoMPaseD, which can effectively guide the selection of proteases to enhance the characterization of specific subsets of the proteome, e.g. based on factors such as protein size, localization or isoelectric point. CoMPaseD is freely available at *https://github.com/MicrobialProteomics/CoMPaseD*.

## Introduction

Small open reading frame (sORF)-encoded proteins (SEP; here defined as proteins ≤ 70 amino acids) have been identified in all kingdoms of life and are involved in various processes within the cell (Storz et al. 2014, Orr et al. 2020). Anticipating their pivotal biological functions, analytical methods for the characterization and detection of this challenging protein class were established and refined during the last years (Klein et al. 2007, Müller et al. 2010, Kubatova et al. 2020, Cassidy, Helbig et al. 2021, Fabre et al. 2021).

A global overview of the repertoire of SEPs present in an organism can be obtained at the different levels of protein synthesis. Dedicated bioinformatic analyses can predict sORFs in the genome (Hanada et al. 2010, Casimiro-Soriguer et al. 2020, Fremin et al. 2022, Yu et al. 2023), albeit only few of these predicted sORFs will actually be translated under at least one condition while the majority might be false-positive hits. Sequencing of ribosome-covered mRNA (Ribo-Seq) can prove the translation of annotated sORFs (Ingolia et al. 2009, Weaver et al. 2019, Venturini et al. 2020, Hadjeras, Heiniger et al. 2023). Finally, proteomic experiments, typically relying on mass spectrometric (MS) methods, allow for the direct detection of SEPs (Cassidy et al. 2016, Kaulich et al. 2020, Fijalkowski et al. 2021, Meier-Credo et al. 2023). In these approaches, either intact proteins are analyzed via MS (top-down) or proteins are enzymatically cleaved into peptides, subsequently identified and used as proxies for their corresponding proteins (bottom-up). While bottom-up proteomics methods are routinely applied, top-down methods have been discussed with special emphasis on small proteins only lately (Cassidy, Kaulich et al. 2021).

Recently, we presented an optimized protocol for the analysis of the small proteome in bacterial samples (Bartel et al. 2020). Besides the enrichment of SEPs by solid-phase columns and adjusted database-search strategies, we also reported that for *Bacillus subtilis*, the application of the protease Lys-C could enhance the validity of the results and provide more robust quantitative data. Likewise, Kaulich *et al*. demonstrated increased sequence coverage and detection of more unique peptides per SEP for *Methanosarcina mazei* when results for samples digested with different proteases were combined (Kaulich et al. 2021). Meanwhile, the (small) proteomes of various species were published involving sample preparation with alternative or multiple proteases (Bland et al. 2014, Fuchs et al. 2021, Petruschke et al. 2021, Hadjeras, Bartel et al. 2023, Hadjeras, Heiniger et al. 2023, Rugen et al. 2023). However, while there are tools such as ProteaseGuru (Miller et al. 2021) available that facilitate the selection of the optimal protease or protease combination to analyze a particular protein, similar tools are not available for experiments targeting the whole proteome or a specific subset of proteins. For such experiments, the decision to apply a particular combination of proteases is usually driven by assumptions or the user’s experience and, thus, not always the most suitable combination is chosen.

In this manuscript, we describe a protease score designed to systematically evaluate the outcome of proteomic experiments with multiple proteases for assessing proteome coverage, sequence coverage and peptide identification rate. Additionally, we present CoMPaseD (Comparison of Multiple Protease  Digestions), a Python-based implementation of an algorithm that can predict the value of this score. As proof of principle, we evaluated CoMPaseD-predicted and experimentally-derived scores for five different proteases (Trypsin, Lys-C, chymotrypsin, Glu-C, and LysArgiNase) and their combined application in the study of the small proteomes from *B. subtilis* and *M. mazei*.

## Materials and methods

### Benchmark Datasets

We tested CoMPaseD against two distinct setups representing different workflows and experimental goals: The first experiment aimed at a comprehensive proteome analysis, including the detection of small proteins encoded by annotated sORFs. For this purpose, the Gram-positive model bacterium *B. subtilis*, strain 168, was grown to the early stationary phase (optical density of 4.0 at 600 nm) in lysogeny broth at 37°C with 180 rpm orbital shaking in a water bath. Proteins were extracted from the cells by ultrasonication, followed by in-solution digestion with trypsin, Lys-C, chymotrypsin, Glu-C, or LysArgiNase. No enrichment for small proteins was performed. After purification, peptides were subjected to LC-MS measurements with CID-fragmentation on an Orbitrap Elite instrument. For details on sample preparation, measurement and data analysis, see Supplementary Material S2. Data for trypsin, Lys-C and chymotrypsin were previously generated in our laboratory (Bartel et al. 2020) and are available from the ProteomeXchange Consortium via the PRIDE partner repository (Vizcaíno et al. 2014) under the identifier PXD017416. Data for Glu-C and LysArgiNase were generated for the present work and are available under the identifier PXD062213. We will refer to this data as *B. subtilis* dataset.

In contrast, the second experiment was designed specifically for the detection of small proteins, including predicted small proteins that are not yet annotated in the reference genome of the methylotrophic methanoarchaeon *M. mazei* strain Gö1. Therefore, the archaeon was cultivated in anaerobic conditions at 37°C under nitrogen starvation and harvested at an optical density of 0.6 at 600 nm. After cell disruption by ultrasonication, the subsequent procedure differed from the first experiment by the application of a workflow tailored to the specific requirements of the analysis of the small proteome. This workflow involved gel-based pre-fractionation of the proteins, digestion from three unstained gel fractions covering the low-molecular weight range, and analysis of the samples on a Q Exactive Plus instrument with HCD fragmentation. Moreover, the search database was supplemented with 1442 sequences of small proteins predicted by transcriptomic mapping of transcription start sites and term-seq (Jäger et al. 2009). This *M. mazei* dataset was generated previously by Kaulich *et al*. (Kaulich et al. 2021) and raw data are available under the PRIDE identifier PXD023921.

### CoMPaseD Workflow

Numerous factors contribute to the observation that typically not all peptides present in a sample are detected in a bottom-up proteomics experiment. These factors include the physicochemical properties of peptides, which can hamper solubility or ionization efficiency; interferences from peptides with similar mass and retention time; or absence from the search space, *e.g*. due to the presence of unexpected modifications or missed cleavage sites (MCs). Additionally, the abundance of proteins, and thus their derived peptides, influences which peptides are detected.

To address these aspects, CoMPaseD employs a Monte-Carlo simulation approach to estimate the efficiency of analyzing the entire proteome or specific subsets of proteins using individual proteases or combinations thereof. The simulation model mirrors the steps of sample generation, digestion with individual proteases, mass-spectrometric detection of the generated peptides and protein inference during data analysis.

Since protein abundances are typically unknown prior to a proteomics experiment but are likely a major determinant of peptide detectability, a random abundance value for each protein in the database is selected from a pool resembling the protein abundance distribution found in bacterial cells. This process is iterated for each simulation. These virtual samples are subsequentially subjected to *in-silico* digestion with the corresponding proteases. MS-measurement is simulated by randomly designating a user-specified number of peptides as identified. Throughout this step, protein abundance serves as a modifier of the detection probability of all peptides from a protein. Optionally, this probability can be further adjusted by the predicted peptide detectability score from our newly trained model (see below). Similar to an actual proteomics experiment, data evaluation involves protein inference from the identified peptides. This information is then used to calculate the protease scores for each virtual sample measurement.

A detailed description of all adjustable parameters, installation procedures, and operational guidance for CoMPaseD is available in Supplementary Material S1.

### CoMPaseD contains three modules that can be run sequentially or independently of each other

The first module is the data preparation module. When executed, this module (i) analyzes the proteins in a user-provided protein database in fasta format, (ii) determines the length of each protein in amino acids, and (iii) groups the proteins based on their length. Further, *in-silico* samples are generated by randomly assigning protein abundance or the absence (i.e. setting the abundance to zero) of a protein for each round of sampling. These abundances are the raw probabilities that a unique peptide, generated by one of the specified proteases, will be selected during the random sampling process. Thus, the probability that a peptide is selected in the first attempt is calculated as its abundance divided by the sum of abundance values for all generated peptides of the proteome. Consequently, peptides derived from the same protein have an identical initial selection probability. The result of the export is saved as a tab-delimited text file, which can be edited by the user to modify protein grouping (for instance, to replace the protein-length dependent classification with cellular localization or biological functions) or to replace simulated protein abundance values with experimental results.

The second module (digestion) automates *in-silico* digestion with several proteases and peptide-to-protein mapping of the results. For this purpose, proteases of interest and the maximal number of expected MCs are extracted from the configuration file, and a series of crux generate-peptides (McIlwain et al. 2014) commands is executed. The mass and length ranges of detectable peptides might also be adjusted in the parameter file. Peptides are then mapped to the proteome, regardless of the cleavage sites of the corresponding protease. Considering only the peptide sequence but not the specificity of protease cleavage is of importance as we demonstrated earlier, that semi-specific cleavage occurs frequently during the analysis of small proteins (Bartel et al. 2020). Therefore, peptides that appear unique under the assumption of specific cleavage may lead to false-positive identifications if semi- or non-specific cleavage is present. This occurs because the expected uniqueness is based on the presence of a cleavage site in one protein, whereas the peptide may originate from a different protein lacking that site. Thus, only peptides that map to exactly one position in the database are treated as unique peptides and are kept for further analysis by default. If required, the filter for uniqueness can be deactivated, and CoMPaseD will keep all peptides, regardless of the number of positions in the database they map to. After annotating each peptide with the protease that generated it, the number of MCs, the assigned protein, and the position within the protein, a combined list of all potentially identifiable peptides is the output of the second module. Notably, identical peptides obtained by multiple proteases are considered unique for each of the proteases (e.g. peptides obtained by tryptic digestion can be the same as those obtained from Lys-C or Arg-C digestion). This resembles an experimental design where the samples from each digestion are analyzed separately, rather than injecting a pooled peptide sample into the MS. Again, the output is stored as a text file that may be used for other purposes or can be edited by the user.

The analysis module is the third part of CoMPaseD. Repeated random sampling of peptides from the pool obtained from digestion with each protease is used to simulate the MS measurement of a sample. For this aim, the assigned protein abundance is transferred to all peptides generated from a protein and used as a weighting factor for the likelihood of detecting a peptide. This module optionally performs the prediction of peptide detectability by DeepMSPeptide (Serrano et al. 2020) with the newly trained model. If this option is enabled, the predicted detectability scores are used to further modify the weighting factor. Prior to random sampling, the total number of peptides required to sample is distributed between peptides with a particular number of MCs based on their expected fraction. Thus, if 10 000 peptides are expected to be identified after digestion with a protease and this protease generates 5% peptides with one MC, 500 peptides would be drawn from the pool of peptides with one MC and 9500 peptides from the pool of fully-cleaved peptides. This enables CoMPaseD to consider the different properties of peptides containing MCs while keeping their frequency among the identified peptides at a realistic value. After sampling, identified proteins are inferred from peptide lists for each sampling replicate and protease scores are calculated for each protein group. In accordance with the “two-unique peptides” rule (Carr et al. 2004), the protease score is additionally calculated assuming this more stringent protein identification criterion. The respective calculated scores and additional results from each sampling are output to the file CoMPaseD_results.tsv, while the mean and standard deviation of the score for each combination and protein group are provided in the file CoMPaseD_results_summary.tsv.

It is noteworthy, that the current implementation, while being able to predict the score for a group of proteins, e.g. small proteins, is not suitable to select a single protein of interest. For such applications, existing programs, such as ProteaseGuru (Miller et al. 2021) should be considered.

### Data Availability

The mass spectrometry proteomics data have been deposited to the ProteomeXchange Consortium via the PRIDE partner repository (Vizcaíno et al. 2014) with the data set identifier PXD062213. Additional raw files were obtained from PRIDE under the identifiers PXD017416 (*B. subtilis*) and PXD023921 (*M. mazei*).

## Results and discussion

### Establishment of a single metric for performance evaluation of bottom-up proteomics experiments

The comparison of different instruments, sample preparation protocols or measurement schemes is a frequent task during method optimization in proteomics. However, the evaluation of experimental outcomes possesses challenges due to inherent ambiguities associated with various metrics. For instance, a higher count of identified proteins might be preferable, but when these proteins are characterized with fewer peptides per protein, the comparability of method performance becomes intricate. To tackle the aforementioned complexities in multiple-protease experiments, we introduce a novel metric, the protease score, aimed at offering a neutral and comprehensive evaluation of data quality.

The principle of this score, however, could be applied to a broader range of research questions, including instrument or sample preparation comparisons.

To construct the protease score, we selected three properties that can easily be obtained from most MS-based proteomics experiments: number of identified proteins, number of identified peptides, and average protein sequence coverage. A high count of identified proteins is indispensable for a comprehensive understanding of a biological sample. Likewise, an increased number of identified peptides reduces the risk of false-positive protein identification and may improve quantification accuracy. Furthermore, a high sequence coverage of identified proteins is imperative for the identification of modified residues and differentiation between closely related proteins with high homology. Notably, the number of identified peptides and protein sequence coverage do not directly correlate for different proteases as the length of generated peptides is dependent on the frequency of cleavage sites. For calculation of the protease score, each property is normalized to the values obtained by tryptic digestion (or any other control condition). After normalization, the metrics are within a comparable numerical range, facilitating aggregation through a weighted geometric mean.

For a group *G* of proteins (where *G* might be a subset of all identified proteins or the complete proteome) and a particular workflow (e.g. protease, method, or protocol), termed *A*, the protease score *S_G,A_* is calculated by:


\begin{eqnarray*}
S_{G,A} & = &\sqrt[ \sum W_{\mathrm{Prot},\,\mathrm{Pep},\,\mathrm{COV}}]{\left(\frac{(N_{\mathrm{Prot}})_{G,A}}{(N_{\mathrm{Prot}})_{G,C}}\right)^{W_{\mathrm{Prot}}}{\rm *}\left(\frac{(N_{\mathrm{Pep}})_{G,A}}{(N_{\mathrm{Pep}})_{G,C}}\right)^{W_{\mathrm{Pep}}}{\rm *}\left(\frac{COV_{G,A}}{COV_{G,C}}\right)^{W_{\mathrm{COV}}}}
\end{eqnarray*}

Where *(N_Prot_)_G,A_* is the number of proteins identified within group *G* by method *A, (N_Pep_)_G,A_* is the number of identified peptides belonging to these proteins and *COV_G,A_* is the average protein sequence coverage of the identified proteins. The same values are calculated for a control workflow, termed *C*, which will usually be tryptic digestion in the case of the comparison of different proteases. If required, either of these properties can be emphasized by modifying its weighting factor (*W_Prot_, W_Pep_* or *W_COV_*). This definition ensures direct comparability between different workflows, where *S_G,A_* values greater than one signify that workflow A outperforms the control workflow.

While the protease score enables neutral comparison of the efficiencies of different proteases or their combinations during the analysis of a (sub-)proteome, predicting this score *in silico* would allow rational optimization of experimental workflows to enhance the detection of specific protein subsets, such as small proteins. To this end, we developed a Monte-Carlo simulation approach specifically designed to predict the protease score. Accurate prediction requires consideration of factors that influence peptide detectability, among which the frequency of MCs plays a central role. The following section describes how MC frequencies were evaluated for eight proteases and incorporated into our predictive model.

### Evaluation of the frequency of MCs for eight proteases

The frequency of MCs for a particular protease may directly influence the properties of the generated peptides. The greater length of peptides with MCs or the often increased hydrophobicity of longer peptides can influence the detection probability of the corresponding proteins. Moreover, when MCs are considered, the detection probabilities of small and large proteins may be differentially affected by the altered peptide frequencies generated from either protein group. Furthermore, considering MCs may enable robust identification of small proteins, which otherwise are identified by only one peptide without MCs as the short length of those proteins typically results in few unique peptides within the analytical window of MS. Thus, the frequencies of MCs should be considered in a model predicting protease efficiencies. For this aim, we aggregated information from several MS-based proteome studies (Supplementary Table 1) (Lawless and Hubbard 2012, Chiva et al. 2014, Guo et al. 2014, Huesgen et al. 2015, Giansanti et al. 2016, Tsiatsiani et al. 2017, Wang et al. 2019, Miller et al. 2021) that applied different proteases and sample preparation protocols. Experimental details are provided in Supplementary Material S2. To avoid information leakage into the prediction model, the two benchmark datasets (*B. subtilis* and *M. mazei*) were excluded from this analysis. Of note, while our analysis provides estimates on the frequency of MCs, it does not indicate the degree of potentially nonspecific cleavage of a particular protease.

**Figure 1.**
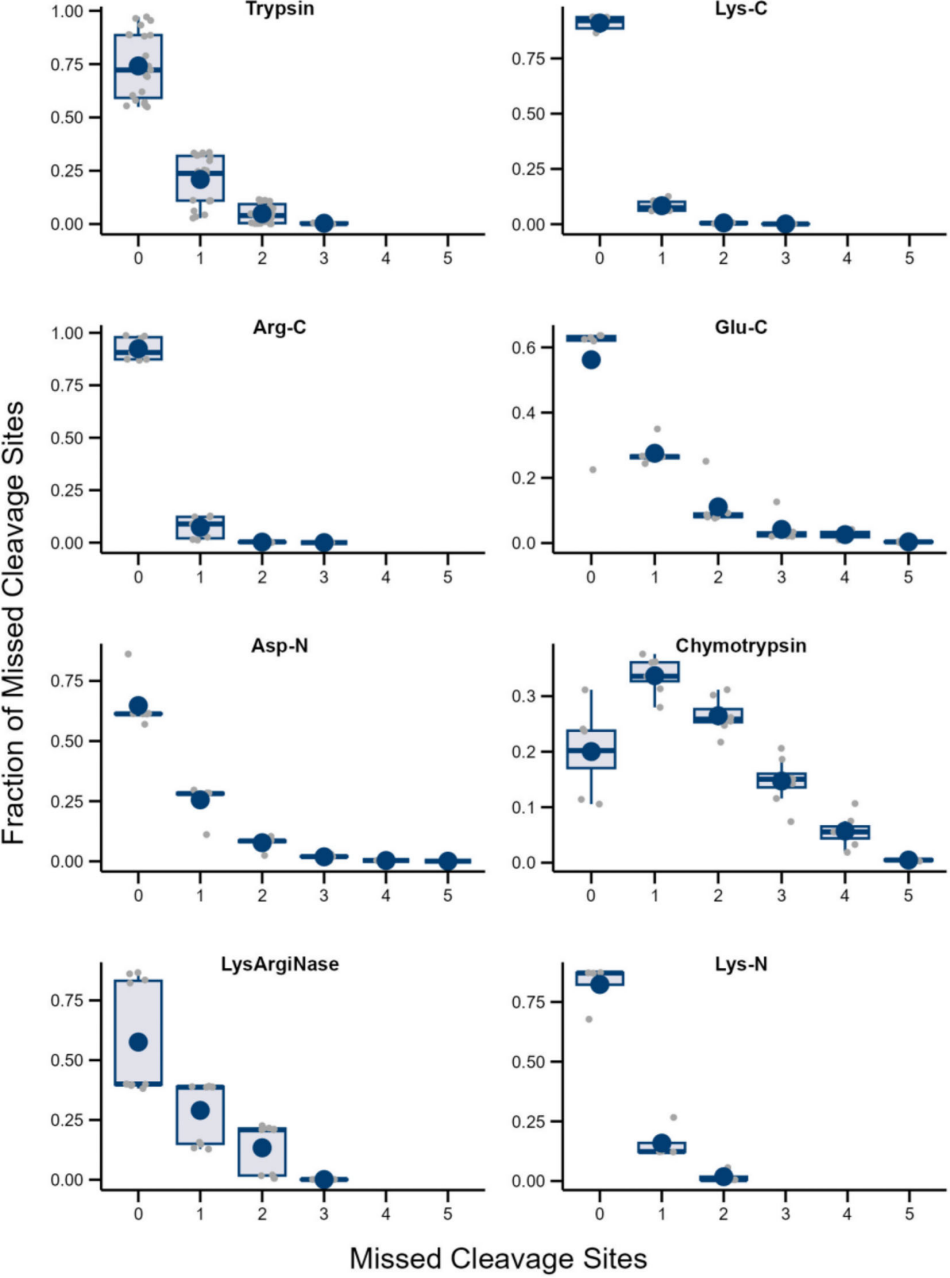
**Distribution of missed cleavage site frequency for different proteases**. Boxplots showing the frequency of identified peptides without or with one to five MCs. Smaller grey dots represent individual datasets analyzed for the corresponding protease, larger blue dots represent mean values and bold horizontal bars represent median values.

The comparison reveals strong differences in the distribution of MCs between proteases (Fig. 1). Notably, cleavage with Arg-C appears almost complete, with only very few peptides containing one or more remaining internal cleavage sites. Contrary, only ∼20% of all peptides generated by chymotrypsin treatment were completely cleaved.

Moreover, a high variance in the fraction of observed peptides with a particular number of MCs was observed for trypsin and, to a similar degree, for LysArgiNase. This observation suggests that the efficiency of different protocols varies widely. While for trypsin we could include data from several, principally different digestion methods, including in-solution digestion and filter-aided sample preparation (FASP), the available data for LysArgiNase was sparse and, consequently, only studies from two groups were included. While Huesgen *et al*. used Hepes buffer at pH 7.5 and digested for 16 h at 37°C (Huesgen et al. 2015), Tsiatsiani *et al*. used a Tris buffer at pH 8.0 and digested for 12 h only (Tsiatsiani et al. 2017), resulting in less complete digestion.

Compared to trypsin, the cleavage efficiency scatters less for Lys-C and Arg-C. Moreover, both proteases produce a higher fraction of peptides without MCs. As Lys-C and Arg-C cleave C-terminal to lysine and arginine residues, respectively, and trypsin cleaves C-terminal to both of these two residues, sequential application of Lys-C and Arg-C instead of trypsin may reduce MCs frequency. Such a protocol would be similar to approaches where trypsin is used only after an initial digestion step with Lys-C (Glatter et al. 2012, Wiśniewski and Mann 2012). While the main aim of concatenated digestion with Lys-C and trypsin is the possibility of applying increased concentrations of denaturing agents, such as urea, the higher overall cleavage efficiency of Lys-C might be a beneficial side effect in these protocols.

In order to prove our hypothesis that the frequency of peptides containing MCs is crucial for determining the optimal combination of proteases, we predicted the protease score for small (≤ 70 amino acids in length) and larger proteins in the *B. subtilis* proteome. We allowed combinations of up to seven proteases and applied either complete digestion (0 MCs) or the mean MC frequencies presented in Fig. 1, which are provided as default settings in CoMPaseD. An equal number of 10 000 peptides identified by each protease was assumed to exclude the influence of differing peptide identification numbers.

We decided to only investigate the effect of MCs on the protease score with an *in-silico* experiment because in experimental data the constrained search space without MCs could lead to false-positive identifications for spectra belonging to MCs-containing peptides and thus may not reveal any disparities. Moreover, proteases with a higher fraction of MCs would be penalized due to the reduced peptide identification numbers, resulting in biased scores. However, the *in-silico* approach assumes that cleavage sites present in small or large proteins have an identical probability of being missed by a particular protease. In practice, the less complex tertiary structure, as well as their shorter length and thus lower number of cleavage sites in small proteins might result in more complete cleavage within the time used for digestion compared to larger proteins.

For small proteins, a significantly smaller protease score was observed when MCs were considered as compared to complete digestion (mean difference: −0.27, paired Student’s t-test *P*-value <10^−16^) while for large proteins the score differed only slightly in the same comparison (mean difference: 0.002, *P*-value <0.005). Notably, the slight score difference observed between control (i.e. 0 MCs) and MC-adjusted predictions for larger proteins is smaller than the experimental variance and thus might hold lesser relevance in practical terms.

Nonetheless, differences between MC-adjusted and control predictions in the ranked score order within combinations of the same number of proteases proved not to be significant (Friedman’s test *P*-values > 0.2 for small and large proteins for all combinations containing 2, 3, 4, 5, 6, or 7 proteases). This is not unexpected as the protease score considers both, the number of identified peptides and, indirectly by the protein sequence coverage, also their length. Thus, the exclusion of peptides with MCs may increase the fraction of peptides belonging to small proteins in the search space but the identifiable peptides would be shorter and thus the average protein sequence coverage would decrease. In our *in-silico* experiment, both single- and multi-protease digestions showed a consistent trend: considering peptides with missed cleavages generally increased sequence coverage for small proteins and slightly decreased it for larger proteins. Specifically, the median sequence coverage increased by 2% for small proteins and decreased by 0.3% for large proteins across all protease combinations (Student’s t-test *P*-value <10^−5^ for both).

### Establishment of a novel prediction model for DeepMSPeptide

Due to the physicochemical properties, differential abundance of various peptides in a sample, and the potential presence of interfering molecules, not all peptides generated by a proteolytic digest are indeed detectable in a proteomics experiment. The semi-stochastic nature of the selection of peptides for fragmentation in data-dependent MS-acquisition schemes further complicates this situation and results in the reproducible detection of only few peptides while other peptides are rarely or never detected. The deep-learning-based tool DeepMSPeptide has been developed to predict detectable peptides based solely on their amino acid sequence (Serrano et al. 2020). However, some properties of the published prediction model compete with its usage in CoMPaseD: Firstly, the training peptides and their detection frequency were obtained from the Global Proteome Machine Database (GPMDB) (Craig et al. 2004), which, owing to its frequent usage in proteomics, mainly contains tryptic peptides. Secondly, the model used binary cross entropy as the loss function during training, which is mostly suitable for classification problems (*i.e*. predicting if a peptide is detectable or not) but inferior for fitting a regression (i.e. predicting how detectable a peptide is).

In order to overcome these limitations, we trained a novel model based on the architecture of DeepMSPeptide. We decided on peptides from the Confetti dataset (Guo et al. 2014) as training data. This dataset aimed at generating a highly complete map of the human HeLa cell proteome by applying multiple protease digestions and therefore contains peptides generated by different proteases. In addition, average HeLa cell protein abundance was obtained from Itzhak *et al*. (Itzhak et al. 2016) and detectability was expressed as each peptide’s spectral counts relative to the expected spectral counts based on protein abundance, assuming well-detectable peptides have values close to one (see Supplementary Material S2 for details). To obtain a more homogeneous distribution of detectability scores than the original DeepMSPeptide model, we changed the loss function to the mean squared error.

**Figure 2.**
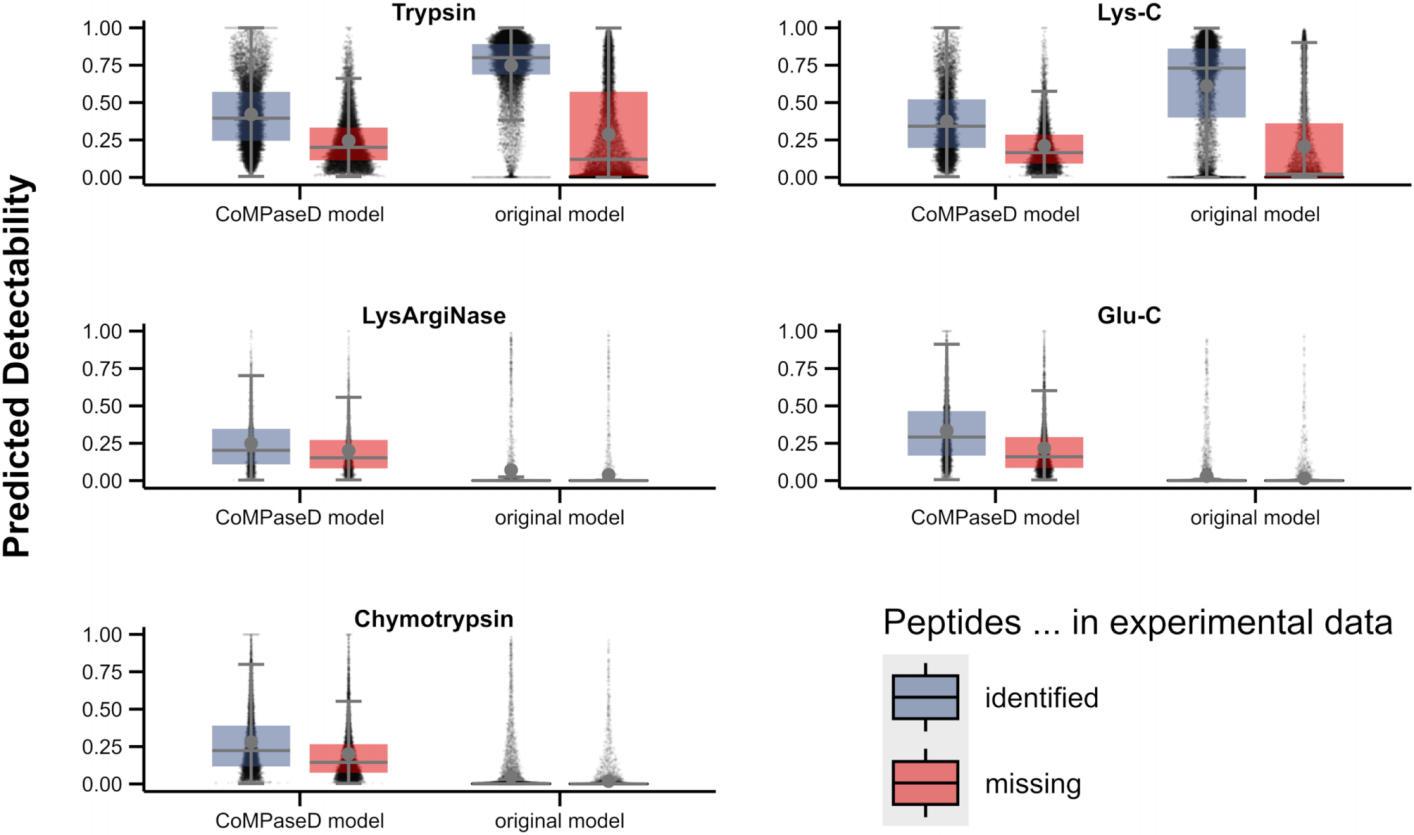
**Comparison of the performance of the original and the CoMPaseD DeepMSPeptide prediction models for different proteases**. Detectability scores for peptides identified (blue) or missing (red) from the *B. subtilis* dataset for five different proteases were predicted either with the original DeepMSPeptide model (original model) or with the DeepMSPeptide model trained with special emphasis on the requirements of CoMPaseD (CoMPaseD model). Black dots in the background represent scores of individual peptides, larger grey dots represent mean values and bold horizontal bars represent median values.

To benchmark our newly trained model, we predicted detectability scores for experimentally identified peptides from our *B. subtilis* dataset. Each identified peptide was randomly complemented by a non-detected peptide originating from the same protein. We assumed that identified peptides should exhibit a higher detectability score than those not detected. As shown in Fig. 2, scores predicted with the original DeepMSPeptide model for peptides with tryptic cleavage sites were in fact higher for identified than for missing peptides. Nonetheless, for some identified peptides, the original model predicted a score close to zero. In particular, a higher density of black dots for detected peptides close to the baseline compared to slightly higher detectability scores can be observed for trypsin in the original model. For Lys-C, cleaving C-terminal of lysine residues, a distribution of detectability scores similar to those of trypsin was observed due to the shared cleavage side of these two proteases. However, for peptides generated by digestion with LysArgiNase, Glu-C, and chymotrypsin, the original model predicted low scores independently of whether these peptides were experimentally detectable.

In contrast, for our novel specialized model, termed CoMPaseD model from here on, the scores were more evenly distributed across the range for both detectable and non-detectable peptides. While this ensures a better correlation between the predicted scores and overall peptide detectability, it also results in higher scores for some missing peptides compared to the original DeepMSPeptide model. Nonetheless, on average, detectable peptides received higher scores than their non-detectable counterparts across all tested proteases, indicating that the CoMPaseD model remains effective at distinguishing detectable peptides, albeit with a less pronounced separation for peptides with tryptic cleavage sites. Notably, LysArgiNase was not used as an enzyme in the training data (nor Lys-N, which shares the cleavage site N-terminal to lysine residues with LysArgiNase). Still, the scores predicted with the CoMPaseD model for LysArgiNase-derived peptides in the test dataset were in a similar range as those for the other proteases. This indicates that the CoMPaseD model can better generalize for enzymes with differing cleavage sites and thus seems more suitable for usage with CoMPaseD compared to the original DeepMSPeptide model.

### CoMPaseD protease score prediction for the analysis of small proteins

We tested the accuracy of our score prediction tool against two different experimental setups: The first experiment was the direct in-solution digestion of a cellular *B. subtilis* protein extract. Five aliquots of this extract were digested in three replicates, each with a different protease (trypsin, Lys-C, chymotrypsin, Glu-C, or LysArgiNase). The resulting peptide mixtures were then separated by reversed-phase chromatography and individually measured with an Orbitrap Elite MS instrument with collision-induced dissociation (CID) fragmentation. Such an experimental setup might be typical when the aim is complete coverage of the proteome or quantification of proteins.

Data for the second experiment were taken from Kaulich *et al*. (Kaulich et al. 2021) and differed from the first by application of a gel-based protein fractionation aiming at the enrichment of small proteins. Proteins below 20 kDa from the methanogenic archaeon *M. mazei* were digested directly in the gel matrix with the same five proteases as used in the first experiment. Peptide mixtures from individual gel pieces were then chromatographically separated and measured on a Q Exactive Plus instrument with higher-energy collisional dissociation (HCD) fragmentation. Moreover, the database used for the analysis of the samples was supplemented with 1442 sequences of predicted small proteins (i.e. 30% of the entries in the final database). This approach is tailored to the analysis of the small proteome. Notably, due to the study of only one condition, the vast majority of the predicted small proteins should be undetectable as they might not be expressed under this condition or be false-positive predictions.

From the resulting MS data, the protease scores for small proteins up to a length of 70 amino acids were calculated for each sample and all possible protease combinations in a replicate-wise manner, and these scores were compared to CoMPaseD-predicted scores. The fraction of undetectable small proteins was set to 45% for *B. subtilis*, which reflects a typical proteome coverage observed in such experiments, while it was set to 98% for *M. mazei* to compensate for the large number of predicted small proteins in the database, which, most likely, will not be expressed under the studied conditions. To reduce the influence of differing peptide identification numbers, we assumed 10 000 peptides identified upon digestion with each protease.

**Figure 3.**
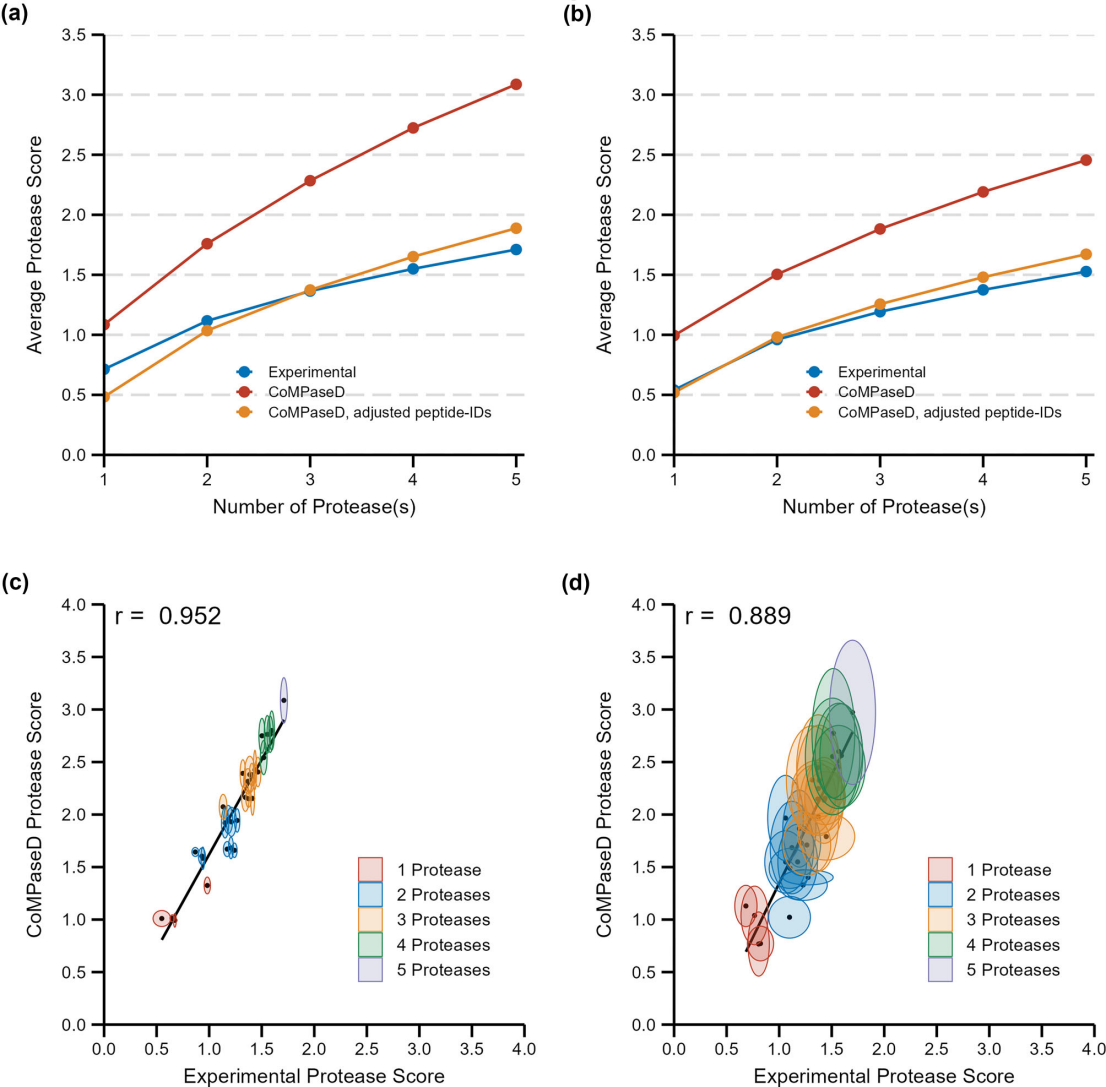
**Comparison and correlation analysis of protease scores**. Comparison between predicted (red) and experimentally derived (blue) protease scores for small (a) and large (b) proteins in *B. subtilis*, considering combinations of one to five proteases. Additional predictions were made for (a) and (b) using the average experimental peptide identification numbers for each protease (orange). Correlation analysis was performed for small proteins from the *B. subtilis* (c) and *M. mazei* (d) datasets, assuming 10 000 identified peptides per protease. Ellipsoid width and height in (c) and (d) represent one unit of standard deviation for three experimental or ten prediction replicates, respectively. Black dots indicate mean values, and Pearson correlation is provided in the upper left corner of the graphs.

As expected, the combination of more proteases results in an increased protease score, and hence data set quality, for small (Fig. 3a, *B. subtilis*; Supplementary Fig. S1a, *M. mazei*) and large proteins (Fig. 3b, *B. subtilis*; Supplementary Fig. S1b, *M. mazei*), which was observed for experimentally derived and CoMPaseD-predicted scores. However, the gain obtained by additional proteases was not linear. In the example of the *B. subtilis* dataset, the difference in the average experimentally observed protease score for small proteins involving two proteases compared to just one was 0.403, while the difference between combinations involving either two or three proteases was only 0.248. Even though the CoMPaseD-predicted scores were generally higher than the experimentally derived scores, this trend of reduced gain by additional proteases was also observed in the predicted scores.

Next, we investigated if the higher-than-expected predicted scores were caused by the fixed number of 10 000 peptides used during prediction. For this purpose, we repeated the CoMPaseD run with identical settings but changed the number of identified peptides to the average value observed in the experimental data. Regardless of whether the summed total number of peptides identified by all five proteases was similar to our previous assumption (50 000), as in the case of *M. mazei* (48 889; Supplementary Fig. S1), or differed to a greater degree, as in *B. subtilis* (36 477; orange line in Fig. 3a and 3b), the predicted scores with these more realistic peptide identification numbers were smaller and closer to the experimental values. These findings suggest that incorporating actual peptide identification numbers or reasonable estimates can fine-tune the predicted scores and help to align them more closely with experimental values, particularly when variations in protease efficiency or digestion protocols affect the number of peptides identified.

In agreement with the observation that parallel digestion with multiple proteases yields greater benefits in experiments aiming at the detection of the small proteome (Bartel et al. 2020, Kaulich et al. 2021), the advantage gained from additional proteases was more pronounced for small proteins compared to large ones, as observed in both, experimental and CoMPaseD-predicted, scores.

As the purpose of CoMPaseD is to determine the protease combination for a particular experimental setup most suitable for the proteomic analysis of small proteins, we also investigated the correlation between CoMPaseD-predicted and experimental scores. Despite assuming a fixed count of 10 000 peptide identifications per protease for score prediction, both score types exhibited strong Pearson correlations of 0.952 for *B. subtilis* (Fig. 3c) and 0.889 for *M. mazei* (Fig. 3d). This correlation further improved when the number of peptides per protease was adjusted according to the observed peptide identification counts (see Supplementary Information S2 for details).

Both tested datasets exhibit differing levels of variability, likely originating from the differences in experimental design. The lower variance between replicates in the *B. subtilis* data allows for clear identification of optimal protease combinations for any defined number of proteases. In contrast, the higher variance in the *M. mazei* dataset results in closely grouped experimental scores, with similarly grouped predicted scores, preventing a definitive conclusion on the best protease combination for this dataset. These results demonstrate that CoMPaseD can predict protease scores for small protein-enriched and non-enriched samples with reasonable accuracy. Interestingly, both predicted and determined scores were less stable between replicates for the small proteome than for larger proteins (Supplementary Fig. S3). This is in accordance with the observation that small proteins are typically less reproducibly identified and emphasizes the particular importance of repeated measurements for small protein analyses. Such samples might be technical or biological replicates (Hadjeras, Bartel et al. 2023), different biological conditions (Bartel et al. 2020) or even the inclusion of multiple different enrichment strategies (Petruschke et al. 2020).

## Discussion

The utilization of alternative proteases, as well as the combination of multiple proteases in independently digested and analyzed samples, has been widely applied in the study of small proteomes. However, there has been a notable lack of tools to aid in the selection of the most suitable protease combinations for optimal proteomic results. To address this gap, we developed the protease score, which allows for an unbiased comparison of proteomics results, and created CoMPaseD for the *in silico* prediction of this score.

The development of CoMPaseD required the determination of baseline missed cleavage frequencies for various proteases and the training of a novel, protease-independent prediction model for peptide detectability, which can be used with the existing DeepMSPeptide framework. We believe that the advances made for CoMPaseD will be useful for future studies by themselves: thus, the principle of the protease score may also be applied for the neutral comparison of different protocols or instrumentation, as it only requires the results of different proteomics measurements. Similarly, the novel DeepMSPeptide model might be useful for the prediction of suitable peptide candidates for PRM assays when alternative proteases instead of trypsin need to be applied. This might, for instance, be the case in the study of modification sites with no tryptic cleavage site in proximity.

Besides the analysis of small proteins, alternative proteases and CoMPaseD-based prediction of optimal protease combinations may also be applied to other applications such as the study of sub-proteomes based on cellular localization (see Supplementary Material S2), particular species within metaproteomic samples, or enhanced coverage of specific functional pathways.

In the field of metaproteomics, unique peptides are rarely detected, and the large number of shared peptides typically results in the formation of protein groups rather than the identification of individual proteins. CoMPaseD supports such a mode. Sequence coverage and the number of peptides per protein are calculated individually for each member of a protein group, and the median values are then used for protease score calculation. However, in analyses where large protein groups are frequently observed, such as is common in metaproteomics, the original protease score calculation could be adapted to reward smaller protein groups, which would enhance data interpretability. The general workflow of CoMPaseD is closely related to experimental workflows. While Monte-Carlo simulations had been used in data evaluation (Mitra et al. 2019), for prediction of exact protein masses (Kilpatrick 2019) and several other applications (Kou et al. 2019, Xu et al. 2020), to the best of our knowledge, this is the first attempt to build a Monte-Carlo simulation model, which targets the quality of a proteomics experiment in advance. Due to the graphical interface of CoMPaseD, which is designed from the perspective of a proteomics researcher, the program can be applied by scientists who are unfamiliar with programming languages.

Recently, the requirements, potentials and risks of an integrated machine-learning model of proteomics experiments have been discussed broadly (Neely et al. 2023). While such a model has not yet been published, CoMPaseD could perhaps benefit from these approaches in future. Thus, for example, protein abundance modelling could be improved by integrating predictions of protein complex formation, assuming defined stoichiometric ratios between member proteins. Likewise, empirical protease cleavage rules could be replaced by the prediction of cleavage sites using a deep-learning model, and peptide sampling could become more realistic when predicted chromatographic separation and co-elution of peptides are considered. However, such attempts would be accompanied by increased requirements on computational power and it is unclear to what extent they could improve prediction accuracy.

## Conclusion

Several small proteomes (Bartel et al. 2020, Fuchs et al. 2021, Petruschke et al. 2021, Kaulich et al. 2021, Hadjeras, Bartel et al. 2023, Meier-Credo et al. 2023) have been published in recent years, and digestion with alternative proteases has frequently been employed in these investigations. With this manuscript, we present CoMPaseD, a software tool to predict the protease combinations that are most suitable for a particular experimental goal. To build this program on a solid theoretical background, we determined baseline missed cleavage frequencies for numerous proteases, generated a novel model for the prediction of peptide detectability by DeepMSPeptide and introduced the protease score, a unified metric for evaluation of result quality of MS-based proteomics experiments. We demonstrated that CoMPaseD can predict optimal combinations of proteases for experiments involving the parallel digestion and measurement of a sample with different proteases, independent of the organism, sample preparation, or the MS measurement. We anticipate that further studies of small proteomes with alternative proteases will be conducted in future and that CoMPaseD could support the authors in selecting optimal protease combinations.

## Supplementary Material

uqaf043_Supplemental_File
