# Supplementary material for: CoMPaseD: advanced planning of proteomic experiments aiming to identify small proteins
Source: Microlife. 2026 Jan 6;7:uqaf043. doi: 10.1093/femsml/uqaf043 (PMC12802878; doi:10.1093/femsml/uqaf043)
Supplement: uqaf043_Supplemental_File [file uqaf043_supplemental_file.docx]

**Supplemental Information**

**CoMPaseD: Advanced Planning of Proteomic Experiments Aiming to Identify Small Proteins.**

Jürgen Bartel^1^, Philipp T. Kaulich^2^, Borja Ferrero-Bordera^1,3^, Rick Gelhausen^4^, Rolf Backofen^4,5^, Andreas Tholey^2^, and Sandra Maaß^1,*^

^1^ Department of Microbial Proteomics, Institute of Microbiology, Center of Functional Genomics of Microbes, University of Greifswald, 17489 Greifswald, Germany

^2^ Systematic Proteome Research & Bioanalytics, Institute for Experimental Medicine, Christian-Albrechts-Universität zu Kiel, 24118 Kiel, Germany

^3^ Department of Molecular Chronobiology, Institute of Medical Psychology, Medical Faculty, LMU Munich, 80336 Munich, Germany

^4^ Bioinformatics Group, Department of Computer Science, University of Freiburg, 79110 Freiburg, Germany

^5^ Signalling Research Centre CIBSS, University of Freiburg, Schaenzlestr. 18, 79104 Freiburg, Germany

* Correspondence: Sandra Maaß – Department of Microbial Proteomics, Institute of Microbiology, University of Greifswald, D-17489 Greifswald, Germany; orcid.org/ 0000-0002-6573-1088; Phone: +49-3834-420-5921; Email: Sandra.maass@uni-greifswald.de; Fax: +49-3834-420-5902.

Table of contents

Supplemental Material S1.

CoMPaseD Program Description and Operation Manual.

Supplemental Material S2.

Experimental Methods.

Supplemental Material S3.

Protease Score Correlation and Peptide Identification Adjustments.

Supplemental Figure S1).

Comparison of Protease Scores in *M. mazei*.

Supplemental Figure S2.

Correlation of Predicted and Experimentally Derived Protease Scores for Small Proteins After Adjustment of the Number of Identified Peptides.

Supplemental Figure S3.

Correlation of Predicted and Experimentally Derived Protease Scores for Large Proteins in *B. subtilis*.

Supplemental Figure S4.

Correlation of Predicted and Experimentally Derived Protease Scores for Different *B. subtilis* Subproteomes.

Supplemental Figure S5.

Correlation of Predicted and Experimentally Derived Protease Scores for Acidic, Neutral and Basic Proteins in *B. subtilis*.

Supplemental Table S1.

Studies Analysed for Evaluation of the Frequency of Missed Cleavage Sites for Various Proteolytic Enzymes.

Supplemental Table S2.

Search Enzyme Definitions as Used During Database Searches of the *B. subtilis* and *M. mazei* Datasets.

**Supplemental Material S1**

**CoMPaseD Program Description and Operation Manual.**

*Installation:*

CoMPaseD (Comparison of Multiple Protease Digestions) is written in Python 3 (tested with version ≥3.5 and ≤3.12) and has been tested on Windows (≥ Windows 10) and Linux machines. Installation and program start should be done via the command prompt. Configuring and running CoMPaseD analyses is possible via the command prompt or a graphical interface.

To run the program, several Python packages and the crux toolkit (McIlwain *et al.*, 2014) need to be available on the computer. The Python packages with suitable version numbers are listed in requirements.txt and may be installed (preferably within an active virtual environment; refer to the Python documentation for further information) using the pip package manager by the command:

python -m pip install -r path/to/requirements.txt

Afterwards, the GitHub repository containing CoMPaseD can be cloned into the same virtual environment:

git clone https://github.com/MicrobialProteomics/CoMPaseD

CoMPaseD can then be started by executing *CoMPaseD_gui.py* or *CoMPaseD_CLI.py*. For the command line version, adjust values in the parameter file and run a full analysis by specifying the parameter file as the only argument or a partial analysis by appending the export (-e), digestion (-d) or result analysis flag(s) (-a, this requires running -e and -d before):

python CoMPaseD_CLI.py -p path/to/parameter.param [-e] [-d] [-a]

Several values from the parameter file may be overwritten by command line options. For a complete listing, display the help text with:

python CoMPaseD_CLI.py –help

For the graphical mode, execute *CoMPaseD_gui.py*. Upon the first program start an error message in the configuration tab indicates errors in the configuration file. Adjust the path to the crux executable and save the altered configuration. For subsequent program starts, the configuration tab should be skipped and CoMPaseD shows the analysis parameter tab directly.


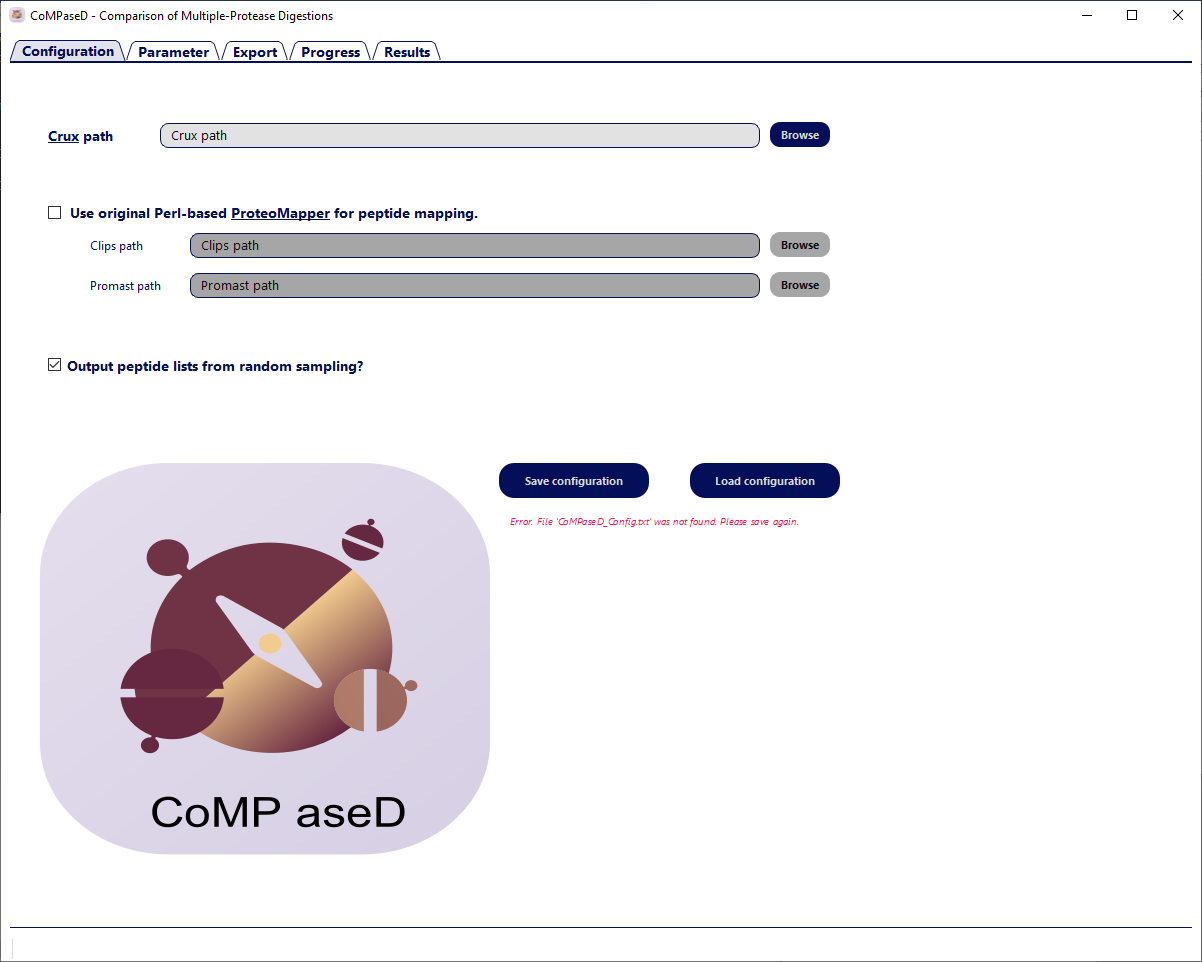


**Initial view upon first program start of CoMPaseD_gui.py.** The red error message will disappear once the path to the Crux executable is properly set and the new configuration is saved. When the checkbox to use original Perl-based ProteoMapper scripts is checked, paths to these scripts must be selected as well and the configuration must be saved again.

*Using CoMPaseD in Graphical Mode:*

For most analyses, the graphical mode will be the preferred option to operate CoMPaseD. All settings can be modified on the parameter tab:

Fasta file:

Select a protein database in *.fasta* format containing all target protein sequences from the proteome(s) of interest. This should be identical to the file used for database searching of the experimental results but must not contain decoy entries.

Output folder:

Specify the storage location of the result files. Missing folders will be created during the analysis. Ensure that CoMPaseD has permission to write and delete files within this folder.


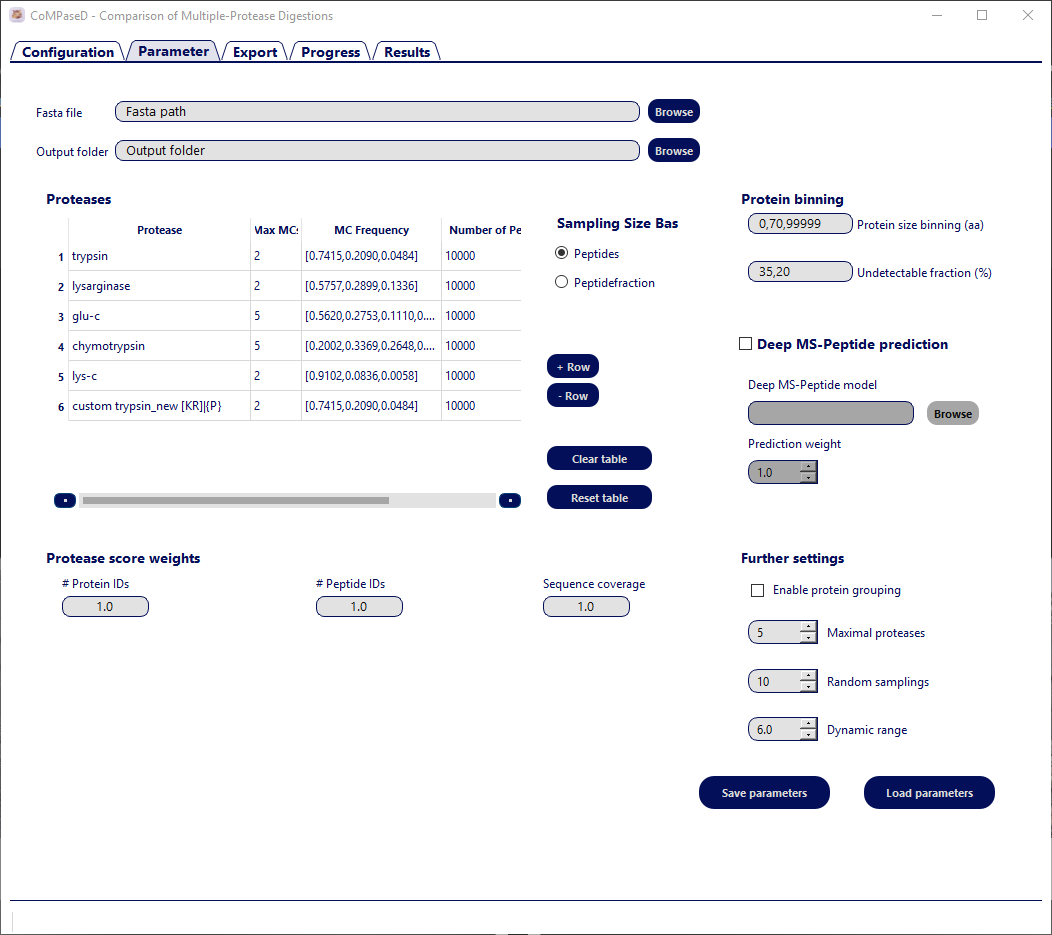


**Parameter tab of *CoMPaseD_gui.py*.** Define parameters for the current analysis.

Protease table:

Specify available proteases in your lab and some additional properties. Protease names in the ‘Protease’ column are limited to those available in the crux toolkit. Currently (Crux version 4.2), these are (underlined proteases do not cleave when the cleavage site is followed by proline):

"trypsin", "trypsin/p", "chymotrypsin", "elastase", "clostripain", “cyanogen-bromide”,

"iodosobenzoate", "proline-endopeptidase", "staph-protease", "asp-n", "lys-c", "lys-n",

"arg-c", "glu-c", "pepsin-a", "elastase-trypsin-chymotrypsin", "lysarginase"

Additionally, custom proteases can be specified by providing the keyword “custom”, followed by a name for the protease and the cleavage specificity. The same syntax as used in the crux toolkit is used to define the protease: Two lists of residues can be specified, either in square brackets to indicate that the residues cause cleavage or in curly braces to indicate prevention of cleavage. Both lists are separated by the vertical bar character (“|”). The first list refers to the amino acid N-terminal of the cleavage site, and the second list to the amino acid C-terminal of the cleavage site. The letter “X” can be used to indicate any amino acid. An example is provided in the figure of the parameter tab as the last line of the protease table.

Specify the maximal number of expected missed cleavage sites (MCs) for each protease in the column ‘Max MCs’ and the frequency of each number of MCs among all identified peptides from this protease in the column ‘MC frequency’, ordered by increasing MCs. The frequencies for all MCs of a protease should sum to 1. Remember to include a value for peptides without MC. As digestion protocols and protease efficiency can vary greatly between labs and enzyme vendors, this parameter should be approximated by the results from earlier experiments in the same group if possible. However, for some commonly used proteases, we aggregated this information from 8 published datasets from different groups (see Supplemental Table S1 for details), and typical values based on this literature review are provided as default settings in CoMPaseD.

Moreover, the expected number of identified unique peptides (alternatively, the fraction of identified peptides of all unique peptides generated) can be set for each protease in the column ‘Number of Peptides’ (alternatively: ‘Fraction of Peptides’). Again, this value depends strongly on the experimental setup and can vary for both, individual proteases and experimental details. Nonetheless, a gross estimate based on results from similar experiments is usually sufficient for the selection of a protease combination.

Protein binning:

Because CoMPaseD was developed to compare the suitability of different proteases or their combinations to detect small proteins, proteins can be grouped based on their length in amino acids in the ‘Protein size binning (aa)’ field, and the protease score is calculated for each group individually. Additionally, a fraction of proteins from each bin can be defined as non-expressed (‘Undetectable fraction (%)’ field). This can be useful, e.g. when working with a database containing in-silico predicted sORFs which mostly will not be expressed *in-vivo*. Setting a high fraction of undetectable proteins for the small protein fraction could then compensate for the bias against large proteins. *Vice versa* for experiments involving the enrichment of small proteins, a greater fraction of the large proteins can be set as undetectable to reflect their depletion. Notably, the grouping scheme can be replaced by user-defined annotations (e.g. cellular localisation) in the export tab using annotation files. These files should be tab-delimited and contain the protein identifier, which appears in the export tab in a column with the header ‘Identifier’.

DeepMSPeptide settings:

Enable or disable the prediction of peptide detectability by DeepMSPeptide (Serrano *et al.*, 2020). If enabled, the weighting factor for each peptide during random sampling will be modified accordingly. The original DeepMSPeptide model was designed to provide a sharp cutoff between detectable and non-detectable peptides and was trained on data from The Global Proteome Machine Database (GPMDB) (Craig *et al.*, 2004), which, owing to the frequent usage of trypsin in MS experiments, mainly contains tryptic peptides. Therefore we trained an alternative model based on the peptides detected within the Confetti dataset (Guo *et al.*, 2014). This dataset consists of several measurements of HeLa digestions with various proteases, likely making it more suitable to train a model for the prediction of peptide detectability with different proteases. Both, the original model and the newly trained model are available from the CoMPaseD GitHub repository. The ‘prediction weight’ is a multiplier that defines the importance of peptide detectability relative to the protein/peptide abundance.

Further settings:

To allow unambiguous identification of the proteins, CoMPaseD considers only unique peptides by default. However, for some experimental workflows, it can be advantageous to include shared peptides and group undistinguishable proteins into protein groups. To enable this functionality, the checkbox ‘Enable Protein Grouping’ can be activated. CoMPaseD will calculate the sequence coverage and the number of peptides per protein for each member of a protein group and use the corresponding median values during protease score calculation.

While the protease score increases with additional proteases, due to limited sample amount, instrument availability, etc., it is often not feasible to apply more than a few proteases in practice. If the number of available proteases exceeds the number of possible measurements, the parameter ‘Maximal proteases’ can be used to limit the number of proteases in a combination and thereby reduce calculation time.

The number of ‘Random samplings’ defines the number of repeated predictions of the protease score. These samplings are carried out on individually initialised protein abundances and apply random peptide sampling for each prediction.

Cellular protein abundances follow roughly a distribution function called Generalised Inverse Gaussian (Koziol *et al.*, 2013), and CoMPaseD randomly assigns protein abundances from a value pool with such a distribution spanning 10 orders of magnitude. However, the dynamic range of a proteomics experiment is typically limited. This is reflected by the option ‘Dynamic range’, which reduces the probability of detecting proteins outside the dynamic range. Larger values will result in more proteins identified but with fewer peptides per protein.

Protease score weights:

The protease score is the weighted geometric mean of the number of identified proteins, the number of identified peptides and the average sequence coverage normalised against the same values from tryptic digestion. The weight for each of these metrics can be adjusted here.

Save/Load parameters:

Save current settings to a parameter file or load settings from a parameter file. Parameters will also automatically be saved when an analysis is started.


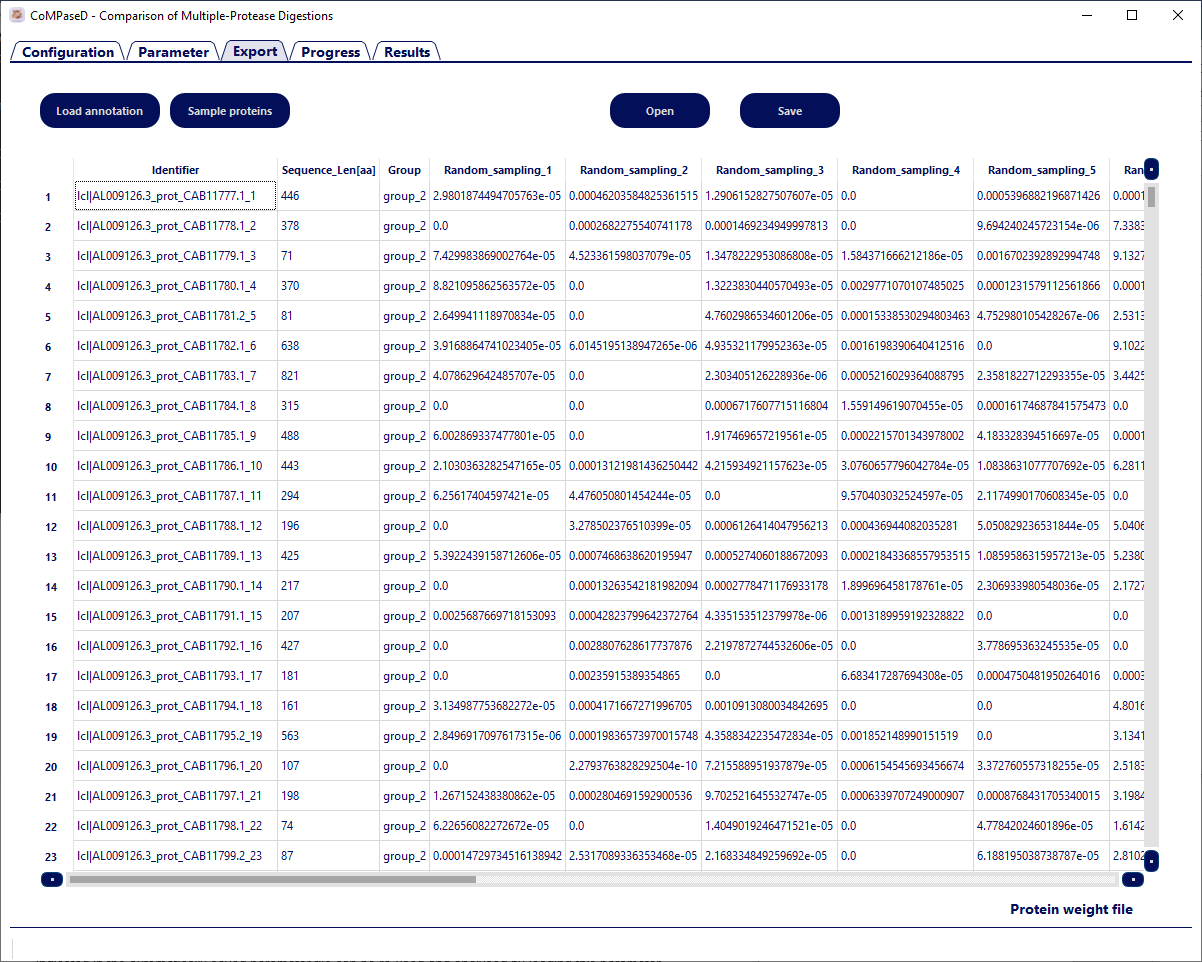


**Export tab of *CoMPaseD_gui.py*.**

Once all settings are made, switching to the export tab will generate a table containing one row per protein in the *.fasta* file and additional columns with randomly initialised abundance values for each protein for each round of random sampling. Abundance values of zero indicate proteins that are excluded as not expressed in the corresponding sampling round.

This table can be manually edited, regenerated with new abundance values by clicking ‘Sample proteins’, or a new protein grouping can be assigned by clicking ‘Load annotation’. Saving this table allows for repeating analyses with different settings, e.g. for the specificity of proteases or the number of identified peptides, while keeping abundance values constant. However, due to the semi-random nature of subsequent peptide sampling, slightly different results are expected for each run, even when parameters are kept constant.

The actual analysis can be started on the progress tab, either by starting a complete analysis (‘Start pipeline’) or by starting separately the digestion or analysis part (‘Start digest’, ‘Start analysis’). The latter can be useful when multiple analyses with the same database and proteases (including the maximal number of missed cleavage sites) are carried out. In such cases, the ‘unique_peptides_table_filtered.tsv’ indicated in the automatically saved parameter file can be reused and analysed by loading this parameter file, adjusting the parameters and starting the analysis directly.

While the analysis is running, progress is printed to the window and parameters cannot be changed.


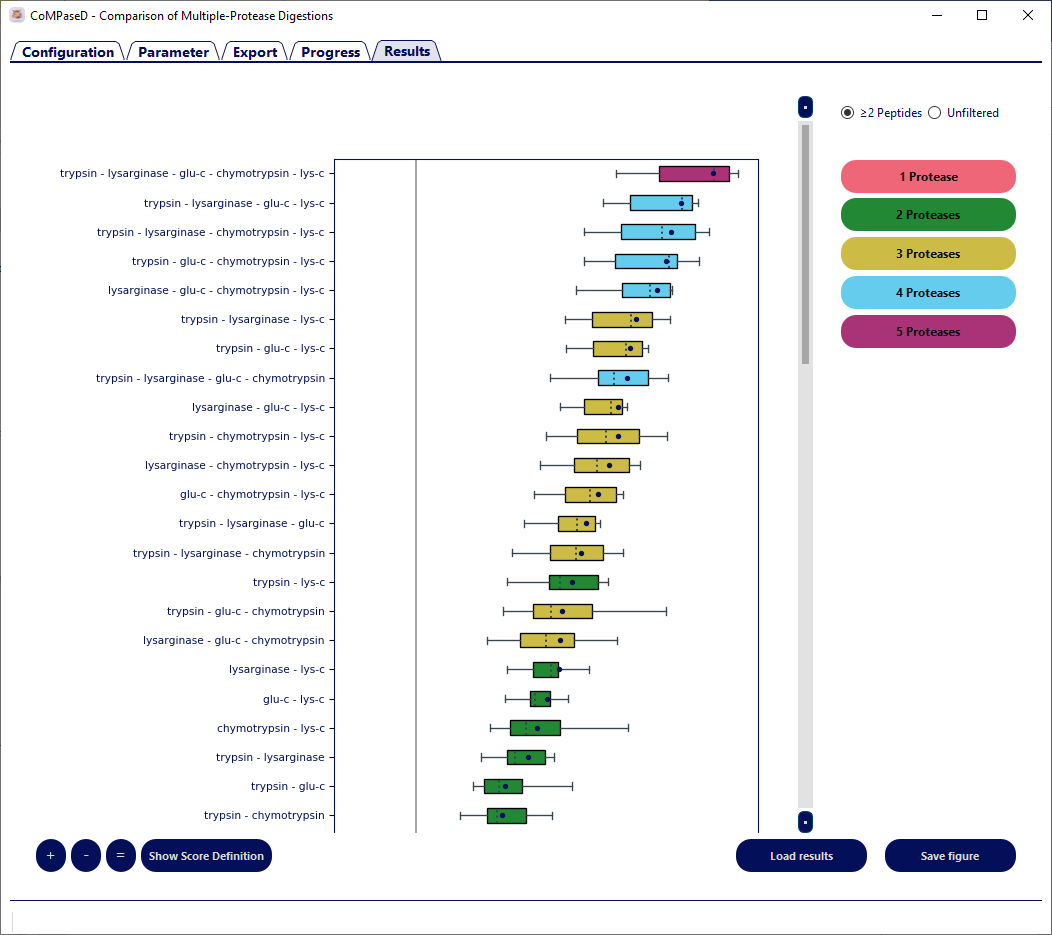


**Result tab of *CoMPaseD_gui.py*.**

After the analysis has finished, the results can be visualised in the results tab. A separate plot is generated for each group defined in the export tab. The radio boxes ‘≥2 Peptides’ and ‘Unfiltered’ toggle between scores that are calculated from all identified proteins or calculated after filtering for at least two unique peptides per protein. The plots can be further customised by removing combinations with a particular number of proteases.

*Advanced CoMPaseD Usage via Command Line:*

For complex analyses or the comparison of different settings, CoMPaseD can be used in a command-line mode by executing CoMPaseD_CLI.py. This also allows the automation of several analyses via shell scripts or batch files. For example, the following commands in a Windows batch file would run an analysis with identical parameters for three different organisms and output the results to folders named ‘Organism_1’, ‘Organism_2’ and ‘Organism_3’:

CoMPaseD_CLI.py -p C:\CoMPaseD.param --out_folder C:\Result\Organism_1 --fasta C:\Organism_1.fasta

CoMPaseD_CLI.py -p C:\CoMPaseD.param --out_folder C:\Result\Organism_2 --fasta C:\Organism_2.fasta

CoMPaseD_CLI.py -p C:\CoMPaseD.param --out_folder C:\Result\Organism_3 --fasta C:\Organism_3.fasta

Similarly, the following command in a Linux shell script would run one analysis with each of the two DeepMSPeptide models using an existing export and digestion file which was specified in the parameter file (note that there is no line break within each command):

python3 /home/user/CoMPaseD/CoMPaseD_CLI.py -p /home/user/CoMPaseD.param -a --DMSP_model /home/user/CoMPaseD/bin/CoMPaseDDMSPModel.h5 --out_folder /home/user/results/confetti_model/

python3 /home/user/CoMPaseD/CoMPaseD_CLI.py -p /home/user/CoMPaseD.param -a --DMSP_model /home/user/CoMPaseD/bin/OriginalDMSPModel.h5 --out_folder /home/user/results/original_model/

Command line options will generally overwrite values in the parameter file and a parameter file with the used values will automatically be saved in the output folder together with the results.

The complete list of options available can be displayed by executing:

CoMPaseD_CLI.py --help

**Supplemental Material S2**

**Experimental Methods.**

*Evaluation of the Frequency of Missed Cleavage Sites for Various Proteolytic Enzymes:*

While proteolytic enzymes used in proteomics typically provide well-defined cleavage motifs, the efficiency of cleavage can vary between proteases, resulting in a variable proportion of missed cleavage sites (MCs). However, due to the effects on the physicochemical properties of the generated peptides, the frequency of MCs will also influence peptide detection properties. To determine a baseline frequency of MCs for eight proteolytic enzymes (trypsin, Lys-C, Arg-C, chymotrypsin, Glu-C, Asp-N, Lys-N and LysArgiNase), we selected eight studies (Supplemental Table 1), which were published between 2012 and 2019, that involved multiple enzymes. The studies were selected based on the comprehensiveness of the dataset, high technical quality, proper experimental design, and the availability of the required data. Information on the number of identified peptides containing a particular number of MCs was extracted. In the case of two studies, this information was not provided and therefore we repeated the database search using MSFragger 3.0 and Philosopher 3.2.9 via the FragPipe 13.0 interface (Kong *et al.*, 2017; Da Veiga Leprevost *et al.*, 2020; Teo *et al.*, 2021). Fractions of MCs were aggregated from at least two independent studies and four samples for each protease to generate boxplots in R (R Core Team, 2022) using the ggplot2 package (Hadley Wickham, 2016).

*Training of a Novel Peptide Detectability Model:*

The machine-learning tool DeepMSPeptide (Serrano *et al.*, 2020) predicts peptide detectability in MS-based experiments by employing a Convolutional Neural Network. For this aim, peptide sequences are encoded by integer conversion and zero-padding and the resulting integer vector is processed by an embedding layer as the first layer of the model. This layer is followed by a dropout layer to prevent over-fitting, two 1D-convolutional layers, a 1D global-max-pooling layer, and a series of dense layers, activation functions and dropout layers, which eventually return the detection probability for the input peptide sequence.

To train a novel, protease-independent model based on this architecture, information from two datasets was combined: The first dataset was the Confetti dataset (Guo *et al.*, 2014), which has been generated by digesting HeLa proteins with a variety of proteases and combinations of proteases to generate a comprehensive proteome map of this human cell line. Peptide sequences and information on spectral counts (SpC) per peptide were extracted. The second dataset was obtained from Itzhak *et al.* and contained the median proportion of total protein mass for individual proteins (m_protein_) in HeLa cells (Itzhak *et al.*, 2016). Peptide sequences were mapped to proteins and the ratio between SpC and m_protein_ was calculated. Linear regression between SpC and m_protein_ was calculated for the top 5% percentile of ratios for unique peptides that were detected in the Confetti dataset with at least 4 spectral counts. Theoretical SpC values (th-SpC) were calculated from the resulting equation for all unique peptides. The ratio between SpC and th-SpC was then used as a measure for peptide detectability based on the assumption that well-detectable peptides should have ratios close to 1 and peptides with low detectability values close to 0.

The resulting data set contained 20 157 peptide sequences belonging to 3230 proteins. For initial testing of the generated model, 2729 peptides belonging to 500 randomly selected proteins were removed from the training data and stored separately. Thus, the training data contained 17 428 peptides belonging to 2730 proteins. The training peptides were used to train a novel model for the prediction of peptide detectability based on the SpC-to-thSpC ratio. Mean squared error was used as the loss function, and different parameters for the individual layers were screened. Correlation of the generated models was tested against the hold-back fraction of peptides and for the final model, training was completed after 309 epochs.

*Digestion with Glu-C or LysArgiNase:*

The newly generated samples of the *B. subtilis* dataset were digested with Glu-C or LysArgiNase. Therefore, aliquots of 20 µg protein were diluted with 50 mM triethylammonium bicarbonate buffer (for LysArgiNase) or 100 mM ammonium bicarbonate buffer (for Glu-C) to a final volume of 100 µl, reduced with 0.5 µmol tris(2-carboxyethyl)phosphine (TCEP) at 65 °C for 45 minutes and alkylated with 1 µmol iodoacetamide at room temperature for 15 minutes in the dark. Digestion was then performed in a temperature-controlled shaker for 12 h at 37 °C using a 1:75 enzyme-to-protein ratio for Glu-C or a 1:25 enzyme-to-protein ratio for LysArgiNase. Upon completion, further proteolytic activity was prevented by adding trifluoroacetic acid to the mixture. The peptides were purified using Pierce C18 Tips (Thermo Fisher Scientific) according to the manufacturer’s protocol. Prior to measurement, samples were reconstituted in solvent A (0.1% (v/v) acetic acid in water), containing retention time calibration peptides (iRT, Biognosys) to monitor the reproducibility of subsequent chromatographic separations (Escher *et al.*, 2012).

*Liquid-Chromatography (LC) and Mass-Spectrometry (MS) of Samples from the B. subtilis Dataset:*

Aliquots containing approx. 1 µg of peptide were loaded with solvent A onto an in-house built 20 cm reversed-phase column (inner diameter 100 µm; outer diameter 360 µm; filled with 3 µm ReproSil-Pur 120 C18-AQ (Dr. Maisch GmbH; Germany)) with an integrated emitter tip. The column was installed at an EASY-nLC 1200 (Thermo Fisher Scientific) system. Separation was achieved by a non-linear gradient of solvent B (0.1% (v/v) acetic acid and 95% (v/v) acetonitrile in water) during 180 minutes, and the eluate was on-line infused in an LTQ Orbitrap Velos Elite (Thermo Fisher Scientific) MS system. In the MS, each cycle consisted of a survey scan in the Orbitrap (300–1700 Th; 60 000 resolution at *m/z* 400; 1×10^6^ predictive automatic gain control target; max. 200 ms injection time; activated lock mass correction), which was followed by up to 20 fragment ion scans. Precursor ions were fragmented by collision-induced dissociation (CID; normalised collision energy of 35) and fragment spectra were detected in the ion trap (mass range dependent on precursor *m/z*; 5×10^3^ predictive automatic gain control; max. 50 ms injection time).

*Database Search and Data Analysis:*

For both benchmark datasets, three biological replicates were generated or downloaded for each protease and searched against a sequence database for *B. subtilis* strain 168 (UniProt proteome ID UP000001570, downloaded on 2021-02-10) or *M. mazei* Gö1 (UniProt proteome ID UP000000595, downloaded on 2019-09-12, supplemented with a list of 1442 SEPs predicted from transcriptomics experiments (Jäger *et al.*, 2009), respectively, using MS-Fragger (v3.1.1.) (Kong *et al.*, 2017). Prior to searching, the databases were supplemented with protein sequences of common laboratory contaminants and Biognosys iRT peptides (Escher *et al.*, 2012). Further, a reversed version of each entry was added. For all proteases, the following parameters were identical: calibrate_mass = 2 (i.e.: on and find optimal parameters); isotope_error = 0/1/2; precursor_mass_mode = selected; num_enzyme_termini = 2; clip_nTerm_M = 1; digest_min_length = 6; digest_max_length = 35; digest_mass_range = 500.0 5000.0. Moreover, up to two MCs and methionine oxidation (+15.994915 Da), deamidation of asparagine or glutamine (+0.984016 Da), conversion of N-terminal glutamic acid or glutamine to pyroglutamic acid (-17.026549 or -18.010565 Da, respectively), and carbamylation at peptide N-termini, lysine, arginine, cysteine, or methionine residues (+43.005814 Da) as variable modifications, as well as static carbamidomethylation (+57.021464 Da) were considered. The search enzyme definition depended on the protease and is listed in Supplemental Table 2. Search output was further analysed by Philosopher (v3.4.13) (Da Veiga Leprevost *et al.*, 2020) using the PeptideProphet / ProteinProphet pipeline as described (Bartel *et al.*, 2020). The complementarity of multiple proteases was investigated by merging their search results from samples of the same biological replicate during PeptideProphet analysis. For the *M. mazei* dataset, the three gel fractions were also merged during this step. Finally, protease scores were calculated (for single proteases or combinations of multiple proteases) from the exported protein and peptide tables using an in-house Python script, which is available at CoMPaseD’s GitHub repository (“CoMPaseD_vs_Experimental_AnalysisScript_subcell.py”).

**Supplemental Material S3**

**Protease Score Correlation and Peptide Identification Adjustments.**

Consistent with the results for *B. subtilis* presented in the main text, the combination of additional proteases in *M. mazei* and the subsequent identification of more peptides leads to an increased protease score for both experimental and *in silico* data. Furthermore, when comparing small and large proteins, the average experimental and predicted scores were consistently higher for small proteins than for large ones for both, *M. mazei* and *B. subtilis* datasets (Supplemental Figure S1). This suggests that alternative proteases offer particular benefits for the analysis of the small proteome.

To test whether the higher predicted scores, compared to experimental ones, were due to the assumed fixed count of 10 000 identified peptides per protease, we repeated this analysis using the average number of experimentally identified peptides for both datasets. For the *B. subtilis* dataset, these values are 13 643 (trypsin), 8010 (Lys-C), 7313 (chymotrypsin), 4909 (Glu-C), and 2602 (LysArgiNase) peptides. For the *M. mazei* dataset, the average numbers are 13 920 (trypsin), 8539 (Lys-C), 7912 (chymotrypsin), 6670 (Glu-C), and 11 848 (LysArgiNase) peptides, respectively. Thus, our assumption aligns well for the *M. mazei* dataset with an average of 9778 ± 3005 peptides (mean ± SD) per protease but differs more for *B. subtilis* for which we identified 7295 ± 4139 peptides on average. Nonetheless, for both datasets this adjustment results in predicted scores that are much closer to the experimental values for small as well as for large proteins.

This analysis was further extended to test whether the correlation between CoMPaseD-predicted and experimental protease scores improved with the adjustment of peptide identification numbers. For small proteins in *B. subtilis*, a higher correlation of 0.971 was observed (Supplemental Figure S2a; compared to 0.952 for a constant 10 000 peptides per protease), while in the case of *M. mazei*, the correlation improved from 0.889 to 0.918 after adjustment (Supplemental Figure S2b). In addition, the more accurate peptide identification numbers resulted in a reduced standard deviation of the protease score prediction for small proteins, bringing the variance closer to the experimental one. Thus, the average relative standard deviation of the predicted scores between the 10 sampling replicates for small proteins from *M. mazei* was 19.3% without adjustment of the peptide identification numbers but 15.7% after adjustment, which is similar to the value of 16.0% observed between the three experimental replicates. A similar trend was observed for small proteins from *B. subtilis*, with average relative standard deviations of 2.9%, 5.9%, and 4.0% for experimental scores, predicted scores, and predicted scores after adjustment, respectively. Interestingly, CoMPaseD captured the different variances between the two experimental setups, indicating that the higher variance in the *M. mazei* dataset is rather an experimental feature.

Next, we used the whole-proteome approach in the *B. subtilis* dataset to test if CoMPaseD can predict protease scores also for different size groups or even completely different groupings.

The protease score prediction for large proteins (> 70 amino acids) depended strongly on the number of identified peptides, and a distinct clustering within combinations with the same number of proteases was observed for the fixed peptide identification numbers (Supplemental Figure S3a). Upon adjustment of the predicted peptide identification numbers to the experimental values, Pearson’s correlation increased to 0.996 (Supplemental Figure S3b) and the distinct pattern within combinations of the same number of proteases disappeared.

We used the same set of sampled peptides to calculate also the protease score for groups of proteins based on the pSortB-predicted cellular localisation (Yu *et al.*, 2010). With a constant 10 000 identified peptides per protease, the scores had Pearson correlations between 0.865 (for cell wall-associated proteins; Supplemental Figure S4a) and 0.926 (for secreted proteins; Supplemental Figure S4g). However, a clear order of the performance of the different protease combinations was only possible when we used the peptide set that was sampled with adjusted identification numbers. Thus, the best binary protease combination for the analysis of proteins from all fractions except the membrane proteins was the combination of Lys-C and trypsin, obtaining predicted scores of up to 1.37 and experimental scores of up to 1.25. However, the second-best combination, chymotrypsin and trypsin, performed significantly worse for all fractions except for membrane proteins where it even obtained a slightly greater predicted score than Lys-C and trypsin (1.33 and 1.29, respectively). The observation of a different order for membrane proteins was made for several protease combinations, indicating that these proteins may require a different set of proteases for efficient analysis.

Finally, we also tested the score prediction for proteins with a different isoelectric point. For this purpose, we split the annotated *B. subtilis* proteins into three groups: acidic proteins with a predicted isoelectric point below 5.5 (Supplemental Figure S5a and b), basic proteins with a predicted isoelectric point above 8.5 (Supplemental Figure S5e and f) and neutral proteins containing the remaining proteins (Supplemental Figure S5c and d). In agreement with the observations made for large proteins and proteins grouped by cellular localisation, the predicted scores clustered mostly by the number of proteases when 10 000 identified peptides per protease were assumed but prediction accuracy improved vastly when we used the adjusted peptide identification numbers. Interestingly, the number of proteases had a much lower importance than the actual choice of the proteases. Therefore, for neutral proteins, the combination of the three proteases chymotrypsin, LysArgiNase and Glu-C did not perform as well as trypsin alone (obtaining a protease score of 1.00 by definition) in both, the predicted (0.90) and experimental scores (0.79).

**Supplemental Figure S1**


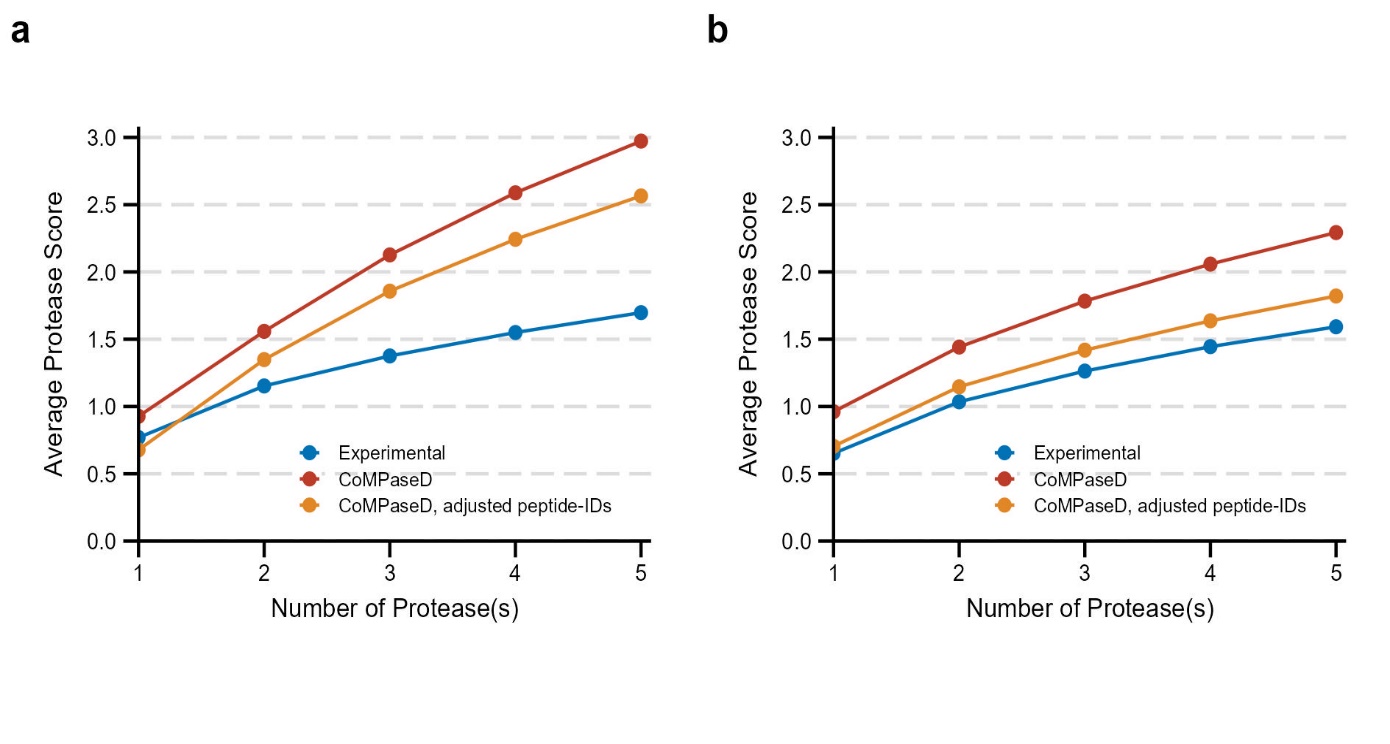


**Supplemental Figure S1: Comparison of Protease Scores in *M. mazei*.** Comparison between predicted (red) and experimentally derived (blue) protease scores for small (a) and large (b) proteins in *M. mazei*, considering combinations of one to five proteases. Additional predictions were made for (a) and (b) using the average experimental peptide identification numbers for each protease (orange).

**Supplemental Figure S2**


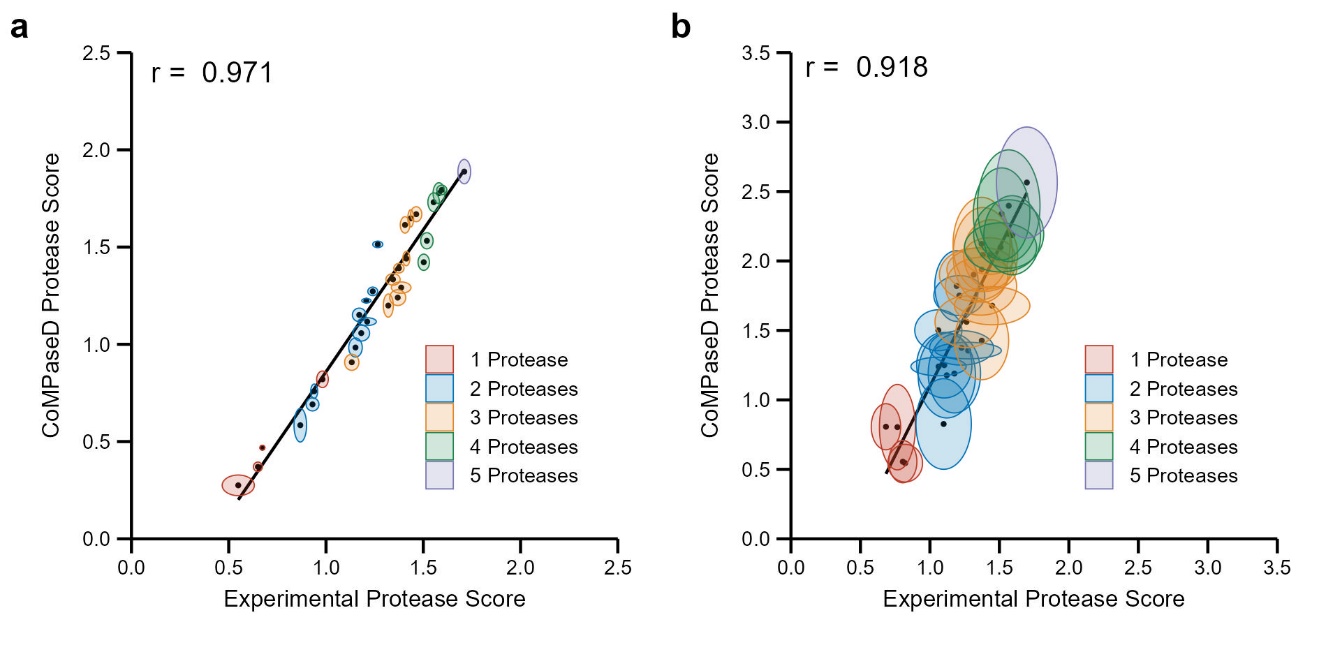


**Supplemental Figure S2: Correlation of Predicted and Experimentally Derived Protease Scores for Small Proteins After Adjustment of the Number of Identified Peptides.** Correlation analysis was performed for small proteins from the *B. subtilis* (a) and *M. mazei* (b) datasets, with peptide identification numbers adjusted to the average experimental value for each protease in both experiments. Ellipsoid width and height represent one unit of standard deviation for three experimental or ten prediction replicates, respectively. Black dots indicate mean values, and Pearson correlation is provided in the upper left corner of the graphs.

**Supplemental Figure S3**


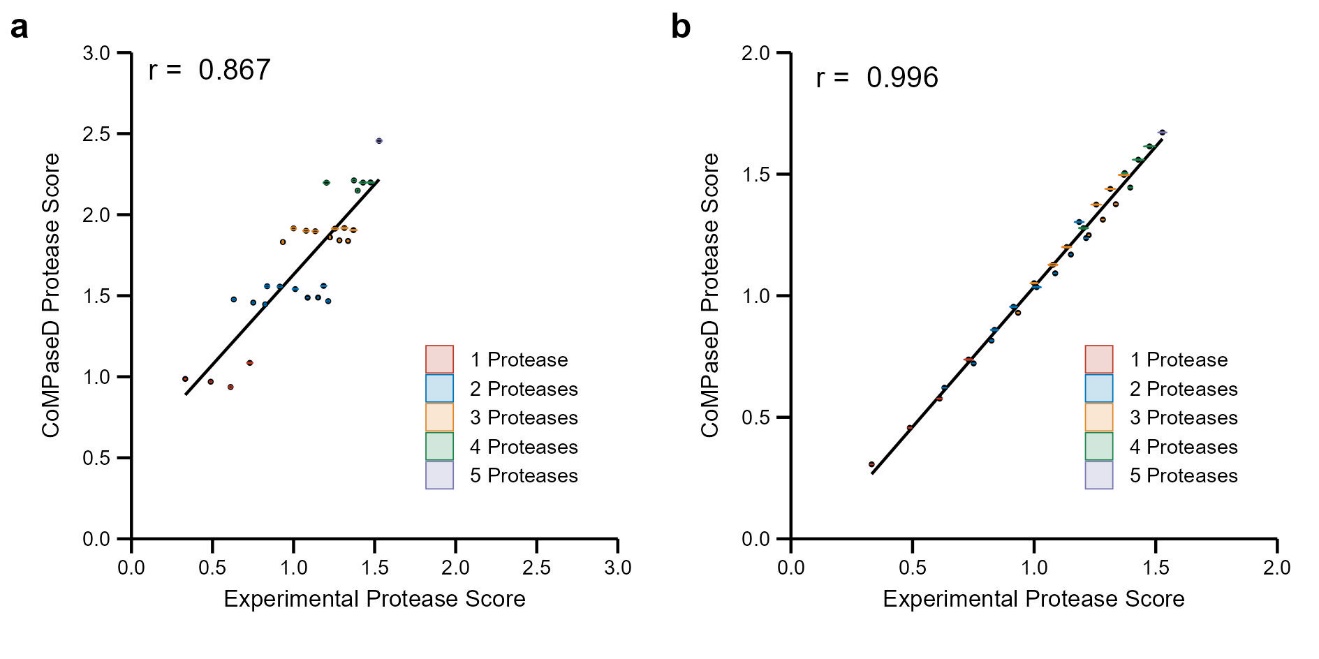


**Supplemental Figure S3:** **Correlation of Predicted and Experimentally Derived Protease Scores for Large Proteins in *B. subtilis*.** Correlation analysis was performed for large proteins (≥ 70 amino acids) from the *B. subtilis* datasets, assuming a constant 10 000 peptides identified per protease (a) or identification numbers adjusted to the average experimental values (b). Ellipsoid width and height represent one unit of standard deviation for three experimental or ten prediction replicates, respectively. Black dots indicate mean values, and Pearson correlation is provided in the upper left corner of the graphs.

**Supplemental Figure S4**


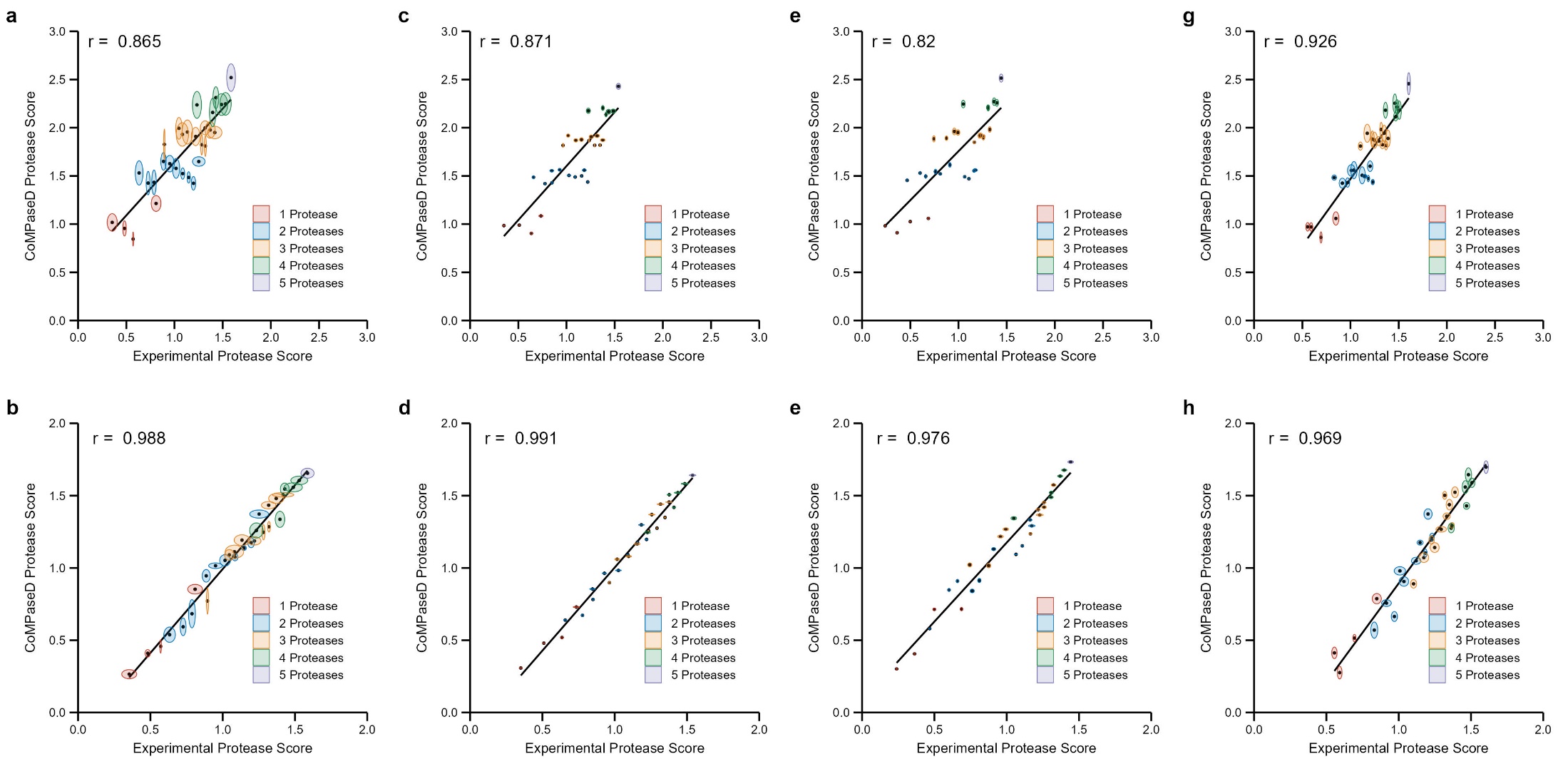


**Supplemental Figure S4****: Correlation of Predicted and Experimentally Derived Protease Scores for Different *B. subtilis* Subproteomes.** Correlation analysis was performed for the *B. subtilis* datasets for cell-wall associated (a, b), cytoplasmatic (c, d), membrane (e, f) and secreted (g, h) proteins. Correlation for proteins with unknown localisation is not shown. Predictions were either performed assuming a constant 10 000 peptides identified by each protease (upper row) or with adjusted peptide identification numbers (lower row). Ellipsoid width and height represent one unit of standard deviation for three experimental or ten prediction replicates, respectively. Black dots indicate mean values and Pearson correlation is provided in the upper left corner of the graphs.


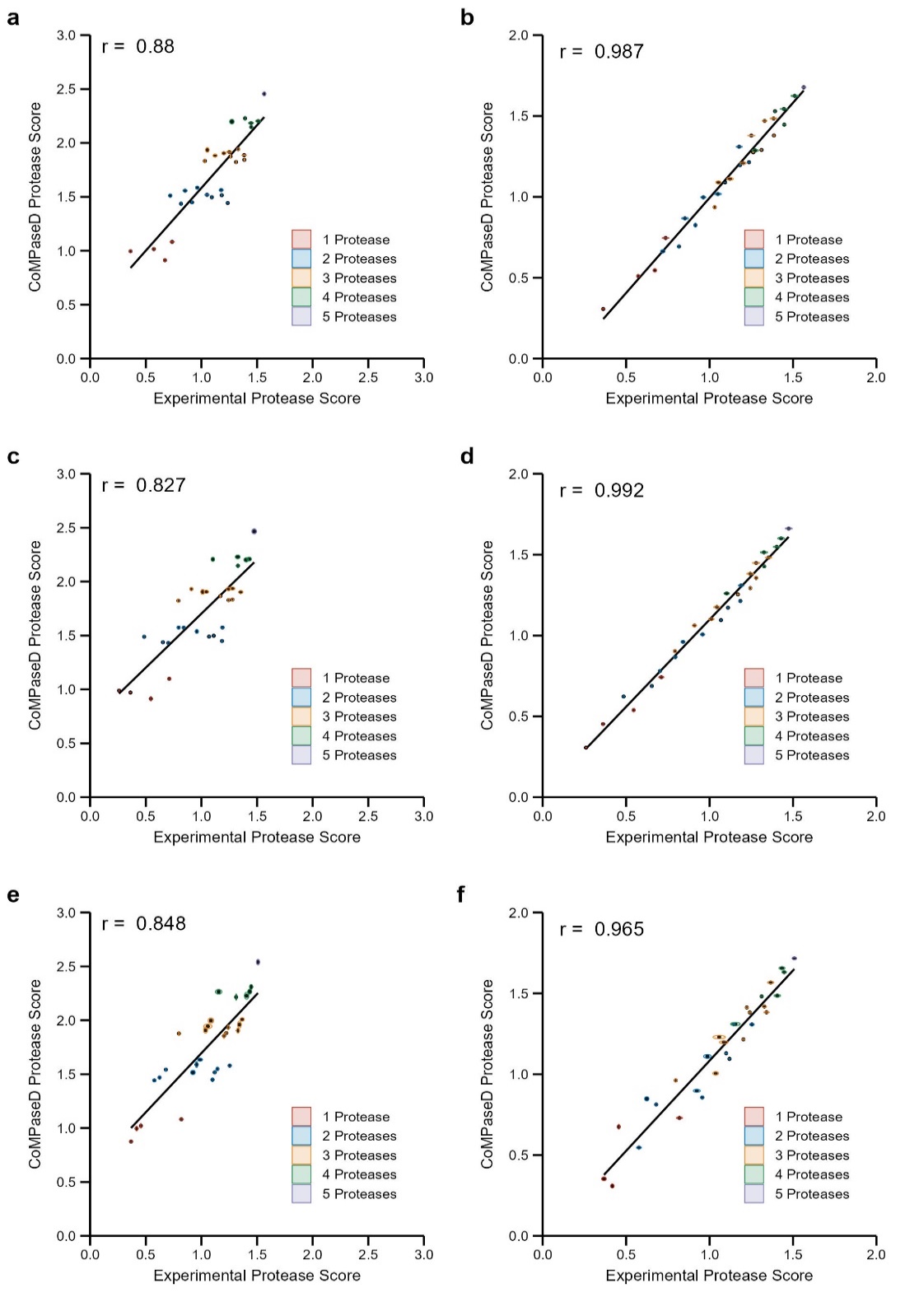
 **Supplemental Figure S5**

**Supplemental Figure S3: Correlation of Predicted and Experimentally Derived Protease Scores for Acidic, Neutral and Basic Proteins in *B. subtilis*.** Correlation analysis was performed for acidic (a, b; calculated isoelectric point below 5.5), neutral (c, d; calculated isoelectric point between 5.5 and 8.5) or basic (e, f; calculated isoelectric point above 8.5) proteins from the *B. subtilis* datasets assuming a constant 10 000 peptides identified per protease (a, c, e) or identification numbers adjusted to the average experimental values (b, d, f). Ellipsoid width and height represent one unit of standard deviation for three experimental or ten prediction replicates, respectively. Black dots indicate mean values and Pearson correlation is provided in the upper left corner of the graphs.

**Supplemental Table S1**

*Studies Analysed for Evaluation of the Frequency of Missed Cleavage Sites for Various Proteolytic Enzymes.*

| **DOI** | **Protocol** | **Organisms** | **Proteases** | **Remarks** |
| --- | --- | --- | --- | --- |
| 10.1021/pr500294d | In-solution and FASP | *E. coli*, spiked with recombinant proteins from bovine, horse, chicken, human, rabbit and pig | Trypsin, Lys-C, Chymotrypsin |  |
| 10.1089/omi.2011.0156 | Various | *S. cervisiae*, *C. elegans*, *D. melongaster* | Trypsin | Meta-study with data from PeptideAtlas |
| 10.1038/nprot.2016.057 | In-solution | *E. coli* | Lys-C, Arg-C, Glu-C, Asp-N, Chymotrypsin, Lys-N |  |
| 10.1038/nmeth.3177 | In-solution | Human breast cancer cell line MDA-MB-231 | Trypsin, LysArgiNase | SILAC labeling |
| 10.1021/acs.jproteome.6b00825 | In-solution | Human lymphoma cell line Jurkat T | Trypsin, LysArgiNase | HCD and ETD; Phosphopeptide enrichment |
| 10.15252/msb.20188503 | In-solution with two-step protease addition | Human tonsil tissue | Trypsin, Lys-C, Arg-C, Glu-C, Asp-N, Chymotrypsin, Lys-N | CID, HCD, ETD, EThcD; hydrophilic SAX |
| 10.1021/acs.jproteome.9b00330 | FASP | Human lymphoma cell line Jurkat T | Lys-C, Arg-C, Glu-C, Asp-N, Chymotrypsin | bRP fractionation |
| 10.1074/mcp.M113.035170 | In-solution | Human cervical cancer cell line HeLa | Arg-C, Glu-C, Asp-N, Chymotrypsin | HCD and CID |

*DOI is underlined where searches were repeated within this study to obtain information on missed cleavage sites.

**Supplemental Table S2**

*Search Enzyme Definitions as Used During Database Searches of the* B. subtilis *and* M. mazei *Datasets.*

|  | **Search enzyme ‘cutafter’** | **Search enzyme ‘butnotafter’** |
| --- | --- | --- |
| Trypsin | KR | - |
| Lys-C | K | - |
| Chymotrypsin | FLWY | P |
| Glu-C | DE | P |
| LysArgiNase | GASPVTCLINDQKEMHFRYW | GASPVTCLINDQEMHFYW |

**References:**

J. Bartel, A.R. Varadarajan, T. Sura, et al. 2020. Optimized Proteomics Workflow for the Detection of Small Proteins. *Journal of proteome research* 19 pp. 4004–4018.

R. Craig, J.P. Cortens, and R.C. Beavis. 2004. Open source system for analyzing, validating, and storing protein identification data. *Journal of proteome research* 3 pp. 1234–42.

F. Da Veiga Leprevost, S.E. Haynes, D.M. Avtonomov, et al. 2020. Philosopher: a versatile toolkit for shotgun proteomics data analysis. *Nature methods* 17 pp. 869–870.

C. Escher, L. Reiter, B. MacLean, et al. 2012. Using iRT, a normalized retention time for more targeted measurement of peptides. *Proteomics* 12 pp. 1111–21.

X. Guo, D.C. Trudgian, A. Lemoff, et al. 2014. Confetti: a multiprotease map of the HeLa proteome for comprehensive proteomics. *Molecular & cellular proteomics : MCP* 13 pp. 1573–84.

Hadley Wickham. 2016. ggplot2: Elegant Graphics for Data Analysis.

D.N. Itzhak, S. Tyanova, J. Cox, et al. 2016. Global, quantitative and dynamic mapping of protein subcellular localization. *eLife* 5.

D. Jäger, C.M. Sharma, J. Thomsen, et al. 2009. Deep sequencing analysis of the Methanosarcina mazei Gö1 transcriptome in response to nitrogen availability. *Proceedings of the National Academy of Sciences of the United States of America* 106 pp. 21878–82.

A.T. Kong, F.V. Leprevost, D.M. Avtonomov, et al. 2017. MSFragger: ultrafast and comprehensive peptide identification in mass spectrometry-based proteomics. *Nature methods* 14 pp. 513–520.

J. Koziol, N. Griffin, F. Long, et al. 2013. On protein abundance distributions in complex mixtures. *Proteome science* 11 p. 5.

S. McIlwain, K. Tamura, A. Kertesz-Farkas, et al. 2014. Crux: rapid open source protein tandem mass spectrometry analysis. *Journal of proteome research* 13 pp. 4488–91.

R Core Team. 2022. R: A Language and Environment for Statistical Computing.

G. Serrano, E. Guruceaga, and V. Segura. 2020. DeepMSPeptide: peptide detectability prediction using deep learning. *Bioinformatics (Oxford, England)* 36 pp. 1279–1280.

G.C. Teo, D.A. Polasky, F. Yu, et al. 2021. Fast Deisotoping Algorithm and Its Implementation in the MSFragger Search Engine. *Journal of proteome research* 20 pp. 498–505.

N.Y. Yu, J.R. Wagner, M.R. Laird, et al. 2010. PSORTb 3.0: improved protein subcellular localization prediction with refined localization subcategories and predictive capabilities for all prokaryotes. *Bioinformatics (Oxford, England)* 26 pp. 1608–15.
